# Correlation of serum cytokines, chemokines, growth factors and enzymes with periodontal disease parameters

**DOI:** 10.1371/journal.pone.0188945

**Published:** 2017-11-30

**Authors:** Jeneen Panezai, Ambereen Ghaffar, Mohammad Altamash, Karl-Gösta Sundqvist, Per-Erik Engström, Anders Larsson

**Affiliations:** 1 Altamash Institute of Dental Medicine, Department of Periodontology, Karachi, Pakistan; 2 Karolinska Institutet, Department of Dental Medicine, Division of Periodontology, Huddinge, Sweden; 3 Habib Medical Centre, Rheumatology Clinic, Karachi, Pakistan, Karachi, Pakistan; 4 Karolinska University Hospital, Department of Laboratory Medicine, Division of Clinical Immunology, Stockholm, Sweden; 5 Uppsala University, Department of Medical Sciences, Uppsala, Sweden; Katholieke Universiteit Leuven Rega Institute for Medical Research, BELGIUM

## Abstract

**Background:**

Periodontal disease (PD) is characterized by inflammatory tissue destruction in tooth supporting apparatus. Many studies indicate that the underlying pathogenesis is in concordance with rheumatoid arthritis (RA) sharing immune-inflammatory events affect both diseases. The aim of this study was to investigate serum cytokines, chemokines, growth factors, enzymes and costimulatory proteins in association with periodontal conditions in PD and RA subjects.

**Materials & methods:**

Periodontal examination was performed in RA (n = 38), PD (n = 38) and healthy subjects (n = 14). Bleeding on probing (BOP) and probing pocket depth (PPD) were measured. Marginal bone loss (MBL) for premolars and molars was measured on digital panoramic radiographs. PD was defined as present if the PPD was ≥5mm in ≥ 3 different sites. Serum samples were collected from all subjects. A multiplex proximity extension assay (PEA) was used to analyze the samples for simultaneous measurement of 92 cytokines. Cytokines with ≥ 60% quantitative results were included.

**Results:**

A significant positive correlation was seen for ST1A1, FGF-19 and NT-3 whereas EN-RAGE, DNER, CX3CL1 and TWEAK associated inversely with BOP, PPD≥ 5mm and MBL but positively with number of teeth. Several CD markers (CD244, CD40, CDCP1, LIF-R, IL-10RA, CD5 and CD6) were found to be associated with BOP, shallow and deep pockets, MBL and number of teeth, either directly or inversely. Most chemokines (CCL8, CX3CL1, CXCL10, CXCL11, CCL11, CCL4, CCL20, CXCL5, CXCL6, and CCL23) were positively associated with number of teeth and some inversely related to MBL (CCL8, CXCL10). Proteins with enzymatic activity (ST1A1, HGF and CASP-8) were directly related to the severity of periodontal conditions and inversely related to number of teeth. Aside from FGF-19, other growth factors were also directly associated with MBL (HGF), number of teeth (VEGF-A, LAP TGF-beta-1) and, inversely to, shallow pockets (LAP TGF-beta-1, TGFA and Beta-NGF). Out of 33 cytokines, 32 associated inversely with shallow pockets, whereas only CD40 associated positively. Associations between cytokines and periodontal parameters in the RA group were comparatively less. Statistical analyses were adjusted for multivariate effects using the Benjamini–Hochberg false discovery rate method.

**Conclusion:**

Systemic inflammatory burden, via known and novel markers, is associated with periodontal conditions in PD and RA subjects. Shallow pockets are not associated with a higher inflammatory state.

## Introduction

In 1944, Menkin discovered ‘factors’ capable of inducing fever and modulating host response to injury [1]. Later experiments led Dumonde to conclude that these factors were released from antigen-activated lymphocytes, leading to the term ‘lymphokines’. Thirty years later Cohen et al discovered that these lymphokines are also produced by non-lymphocytic cell lines and the term ‘cytokines’ was proposed for these factors [2–4].

Today, it is well known that cytokines play a crucial part in the complex interactions that take place in inflammation and immunity in order to maintain health. Although they are produced transiently in health, cytokines can behave in an autocrine, paracrine or endocrine fashion, exerting pleiotropic effects on a large repertoire of cells. [5].Chemokines are cytokines with an inherent property to induce chemotaxis in cells. This allows them to be involved in leukocyte trafficking as well as maturation. Any aberration in the cytokine and chemokine signaling can have detrimental effects leading to pathogenic involvement in inflammatory diseases as well as cancer [6]. In chronic inflammation, chemokines selectively recruit pro-inflammatory cells and maintain them, as required, during the inflammatory response [7]. Periodontal disease (PD) is widely recognized as a disease of chronicity for which no precise reason is known. Bacterial involvement is strongly believed to initiate PD but it is the host response which determines the extent of immuno-inflammatory damage to the periodontal tissues [8]. Cytokines and chemokines are the key effector molecules determining the extent of the inflammatory milieu and tissue destruction. Chemokines also play an important role in synovial inflammation and tissue destruction in rheumatoid arthritis (RA). Some studies have shown that more production of chemokines was associated with early inflammatory events, including leukocytosis and the production of acute phase reactants [9].

Although cytokines are a very crucial biological network, there is still a lack of understanding of this network in periodontal disease [10]. An important step in understanding the role of these biological mediators is to simultaneously study a large number of cytokines to achieve a broader picture of the cytokine network rather than characterize individual cytokine responses in periodontal pathogenesis. The aim of this study was to investigate the association between serum levels of a broad range of cytokines and chemokines in PD and RA subjects in relation to periodontal conditions.

## Material and methods

### Study population

A total number of 90 subjects were included in the present study at the Department of Periodontology, Altamash Institute of Dental Medicine in Karachi, Pakistan, between October 2012 and April 2016. All subjects were adults (≥18 years). All subjects gave their written informed consent to participate in the study. A questionnaire was used to acquire information regarding hospitalization history and the presence of self-reported chronic diseases. Detailed information regarding use of medication, smoking habits, oral hygiene measures, oral health status and past dental history was recorded.

Individuals with osteoarthritis, gout, and a history of treatment for periodontal disease during the last six months and / or treatment with antibiotics in the last three months were excluded.

Thirty eight RA patients (33 females and five males range: 21 to 70 years; mean age ± SD: 46.1 ± 11.9 years) were recruited consecutively from the Rheumatology Clinic in Habib Medical Centre, Karachi. They were diagnosed in accordance with the 2010 ACR / EULAR (American College of Rheumatology and the European League Against Rheumatism) Rheumatoid Arthritis Classification Criteria by the referring rheumatologist (AG) [11]. The criteria comprised of four categories which were joint involvement, serology, acute phase reactants and duration of symptoms. Each of these criterion was applied to patients with presence of obvious clinical synovitis in at least 1 joint. All RA subjects had been receiving disease modifying anti-rheumatic drugs (DMARD).

Thirty eight subjects attending the Department of Periodontology seeking periodontal treatment at the Altamash Institute of Dental Medicine, Karachi, were also included. They suffered from periodontal disease (26 females and 12 males, range: 25 to 64 years; mean age ± SD: 47.4 ± 9.3 years). Periodontal disease was defined as three sites or more with PPD of ≥5mm. Another 14 healthy individuals attending the hospital (nine males and five females, range: 35 to 60 years; mean age ± SD: 44.4 ± 6.6 years) were selected as controls. All controls had clinically healthy periodontium and no systemic disease.

### Periodontal clinical examination

Bleeding on probing (BOP) and periodontal probing depth (PPD) were recorded for all teeth excluding third molars. The indices recorded four sites per tooth. Periodontal disease was defined as three sites or more with PPD of ≥5mm using Goldman-Fox probe (Hu-Friedy, Chicago, IL, USA) [12]. BOP was recorded as local bleeding present within 30 seconds upon probing. Plaque index (PI) was also recorded for four sites per tooth. The scores were recorded for four sites per tooth as a mean percentage for all three parameters.

### Radiographic measurements

A digital extra oral tomography machine (SironaOrthophos 3, Germany) was used to take panoramic radiographs. Radiographs were viewed on a computer screen. Digital measurements were made using SIDEXIS software. One pixel was equal to 0.09mm. Marginal bone loss (MBL) measurements were made as an assessment of the vertical distance from the cementoenamel junction (CEJ) to the most apical portion of the marginal bone. MBL was measured for premolars and molars (excluding third molars). An average value for MBL per tooth was calculated after taking two readings of mesial and distal sides each on digital radiographs.

### Blood sampling

Blood samples were collected from all subjects. The samples were collected in BD Vacutainer™ plastic blood collection tubes (4 mL) without any additives. Blood was allowed to coagulate and tubes were then centrifuged at 1790 x *g* for 10 minutes. The serum was removed and transferred to 2ml storage tubes and were kept frozen at -22°C.

### IgM-RF and ESR measurement

IgM-RF levels were measured using turbidimetric reagents to quantify rheumatoid factor (RF) using an automated clinical chemistry system. The reagent Quantia RF 6K44-01(Abbott Diagnostics, Illinois, USA) comprises of R1 (activation buffer) and R2 which is a suspension of polystyrene latex particles of uniform size coated with human gamma globulin. Upon mixing of the sample with the reagent R1 and R2, agglutination occurs which is then measured by turbidimetry allowing quantitative determination of IgM-RF using Abbott ARCHITECT c8000 system (Abbott Diagnostics). Results are expressed in IU/mL based on the WHO standard [13]. The system was closely monitored by routine practice of running both high and low controls with every batch analyzed. The within-run CV was 0.6% at concentration 54.8 IU/ml and 0.7% at 119.9 IU/ml. The levels of RF < 30 U/ml were considered normal, 30–50 U/ml as low-level positive and values > 50 U/ml as high-level positive. ESR was estimated by using the Westergren method manually (normal range: 0 to 20 mm per hour).

### Anti-CCP detection

BioPlex® 2200 immunoassays was used to analyze anti-CCP levels in the serum samples (BioPlex™ 2200 anti-CCP, Bio-Rad Laboratories, Hercules, CA, USA). This immunoassay method is based on the heterogeneous sets of magnetic beads. Using the BioPlex® 2200 Anti-CCP kit, which is a multiplex flow immunoassay, semi-quantitative detection of IgG antibodies to cyclic citrullinated peptide (CCP) was measured in serum. The system combines a patient sample aliquot, sample diluent, and bead reagent into a reaction vessel and incubates the mixture at 37˚C. After a wash cycle to remove unbound antibody, anti-human IgG conjugated to phycoerythrin is added to the mixture and incubated at 37˚C. Excess conjugate is removed in another cycle and the washed beads are re-suspended on wash buffer. The bead mixture then passes through the detector and the assay identity is determined by the fluorescence embedded in the surface of the bead. The amount of immobilized antibody is determined by the fluorescence of the anti-IgG reporter conjugate. Raw data are collected in relative fluorescence intensity (RFI). The RFI is converted to U/mL using the calibration curve established by the 6 levels of BioPlex 2200 Anti-CCP Calibrators. All samples were run as singletons.

### Ethical approval

The study was approved by the ethics committee of the Altamash institute of Dental Medicine, Karachi, Pakistan (2012-09-26, 2016-09-30) and the Regional Ethical Review Board in Stockholm, Sweden (2016/296-31/1). It was conducted in accordance with the Declaration of Helsinki.

### Proximity Extension Assay (PEA)

Proseek Multiplex Inflammation I (Olink Bioscience, Uppsala, Sweden), was used for conducting PEA, according to the manufacturer's instructions. The panel simultaneously measures 92 biomarkers, as a homogeneous assay, in a 96-well microtiter plate format (http://www.olink.com/products/inflammation/). The samples were assayed as singletons. One microliter (μl) sample was mixed with 3 μl incubation mix that contained pairs of probes (each consisting of a DNA oligonucleotide labelled). This mixture was incubated at 8°C overnight, followed by addition of 96 μl extension mix containing PEA enzyme and PCR reagents which were added and incubated for 5 min at room temperature before transferring the plate to a thermal cycler. In the cycler, the plate underwent 17 cycles of DNA amplification. A 96.96 Dynamic Array IFC (Fluidigm, South San Francisco, CA, USA) was prepared and primed according to the manufacturer's instructions. Using 7.2 μl detection mix and mixing it with 2.8 μl of sample mixture in a new plate, 5 μl of this was loaded into the right side of the primed 96.96 Dynamic Array IFC. On the left side of the 96.96 Dynamic Array IFC, the unique primer pairs for each cytokine were loaded and the protein expression program was run in Fluidigm Biomark reader, in accordance with the instructions. Details regarding data validation, limit of detection (LOD), specificity and reproducibility are available via Olink’s website (http://www.olink.com/data-you-can-trust/validation/). Calibrator curves for correlating the normalized protein expression (NPX) values with actual concentrations can also be found in Olink’s website (http://www.olink.com/proseek-multiplex/inflammation/biomarkers/). The assays were performed blinded without knowledge of clinical data. The assay protocols can be viewed using the link https://protocols.io/view/correlation-of-serum-cytokines-with-periodontal-di-jvkcn4w.

### Statistical analyses

The data was normalized using a Wizard generated by Olink, together with the statistical software GenEx. The assay generated a delta Cq (dcq) value for each data point via Olink Wizard with GenEx software thus normalizing each sample for technical variation in one run. This was followed by normalization between runs through subtraction of the interplate control (IPC) for each assay. For the final step, the values were set relative to a fixed correction factor which is determined by Olink. The generated Normalized Protein eXpression (NPX) unit is on a log2 scale where a larger number represents a higher protein level in the sample, with the background level at around zero. The data used for statistical analysis was reported in NPX units.

Spearman’s rank correlation was performed to analyze the associations between cytokine levels and clinical parameters, respectively. Group wise comparison was not done in order to restrain the occurrence of Type I errors which is common in multiple testing. Instead, all subjects were used as a singular cohort to analyze periodontal correlations with systemic levels of cytokines. To further reduce the risk of false discoveries due to multiple testing, the Benjamini–Hochberg false discovery rate method was used to adjust the p-values [14]. Adjusted p-values less than 0.05 were considered significant which corresponds to an expected false discovery rate of 5%. The calculations were performed using SPSS version 21.0 (SPSS Inc, Chicago, IL, USA). Inclusion of biomarkers was done where ≥ 60% of subjects had detectable levels. In total, 66 cytokines were used for analyses in this study (Table 1).

**Table 1:** Biomarkers* with ≥ 60% of results included in the analyses.

Adenosine Deaminase (ADA)	Interleukin-10 receptor subunit alpha (IL-10RA)
Beta-nerve growth factor (Beta-NGF)	Interleukin-10 receptor subunit beta (IL-10RB)
Caspase 8 (CASP-8)	Interleukin-12 subunit beta (IL-12B)
C-C motif chemokine 2 (CCL2) “MCP-1”	Interleukin-15 receptor subunit alpha (IL-15RA)
C-C motif chemokine 3 (CCL3) “MIP-1 alpha”	Interleukin-18 (IL-18)
C-C motif chemokine 4 (CCL4)	Interleukin-18 receptor 1 (IL-18R1)
C-C motif chemokine 7 (CCL7) “MCP-3”	Latency-associated peptide transforming growth factor beta 1 (LAP TGF-beta-1)
C-C motif chemokine 8 (CCL8) “MCP-2”	Leukemia inhibitory factor receptor (LIF-R)
C-C motif chemokine 11 (CCL11) “Eotaxin”	Macrophage colony-stimulating factor 1 (CSF-1)
C-C motif chemokine 13 (CCL13) “MCP-4”	Matrix metalloproteinase-1 (MMP-1)
C-C motif chemokine 19 (CCL19)	Matrix metalloproteinase-10 (MMP-10)
C-C motif chemokine 20 (CCL20)	Natural killer cell receptor 2B4 (CD244)
C-C motif chemokine 23 (CCL23)	Neurotrophin-3 (NT-3)
C-C motif chemokine 25 (CCL25)	Oncostatin-M (OSM)
C-C motif chemokine 28 (CCL28)	Osteoprotegerin (OPG)
CD40L receptor (CD40)	Programmed cell death 1 ligand 1 (PD-L1)
CUB domain-containing protein 1 (CDCP1)	Protein S100-A12 (EN-RAGE)
C-X-C motif chemokine 1 (CXCL1)	Signaling lymphocytic activation molecule (SLAMF1)
C-X-C motif chemokine 5 (CXCL5)	SIR2-like protein 2 (SIRT2)
C-X-C motif chemokine 6 (CXCL6)	STAM-binding protein (STAMPB)
C-X-C motif chemokine 8 (CXCL8) “IL-8”	Stem cell factor (SCF)
C-X-C motif chemokine 9 (CXCL9)	Sulfotransferase 1A1 (ST1A1)
C-X-C motif chemokine 10 (CXCL10)	T-cell surface glycoprotein CD5 (CD5)
C-X-C motif chemokine 11 (CXCL11)	T cell surface glycoprotein CD6 isoform (CD6)
C-X_3_-C motif chemokine ligand 1(CX3CL1) “Fractalkine”	TNF-beta (TNFB)
Cystatin D (CST5)	TNF-related activation-induced cytokine (TRANCE)
Delta and Notch-like epidermal growth factor-related receptor (DNER)	TNF-related apoptosis-inducing ligand (TRAIL)
Fibroblast growth factor 19 (FGF-19)	Transforming growth factor alpha (TGF-alpha)
Fms-related tyrosine kinase 3 ligand (Flt3L)	Tumor necrosis factor (Ligand) superfamily, member 12 (TWEAK)
Hepatocyte growth factor (HGF)	Tumor necrosis factor ligand superfamily member 14 (TNFSF14)
Interleukin-6 (IL-6)	Tumor necrosis factor receptor superfamily member 9 (TNFRSF9)
Interleukin-7 (IL-7)	Urokinase-type plasminogen activator (uPA)
Interleukin-10 (IL-10)	Vascular endothelial growth factor A (VEGF-A)

*Common synonyms used in Olink inflammation Panel are shown in double inverted commas.

## Results

Two samples (healthy controls) were excluded due to unacceptable technical variations, therefore, the final cohort group consisted of 88 subjects.

### Characteristics of study subjects

Characteristics of study subjects are presented in Tables 2 and 3.

**Table 2:** Characteristics of study group.

Total subjects (n)	90
Mean age distribution in years (range)	46.4 ± 10.1 (21–70)
Females (n, %)	64 (71)
Total subjects with RA (n)	38
Subjects with RA and PD (n, %)	19 (50)
Subjects with RA and without PD (n, %)	19 (50)
Total subjects with PD only (n)	38
Healthy Controls (n)	14

RA = rheumatoid arthritis PD = periodontal disease.

**Table 3:** Characteristics of the rheumatoid arthritis (RA) group.

Characteristic	RA without PD (n = 19)	RA with PD (n = 19)
Years since diagnosis	8.7	8.1
IgM-RF (≥30 IU/mL)	117.5	168.7
ESR (mm/hr)	49.8	54.8
Anti-CCP (≥ 3 U/mL)	251.7	286.1

Values are shown as mean ± SD.

IU = international units

Anti-CCP values are expressed as arbitrary units (U/mL)

PD = periodontal disease

### Periodontal parameters

The periodontal variables for all study groups are presented in Table 4.

**Table 4:** Clinical (BOP, PPD 3-<5mm, PPD ≥5mm, number of teeth) and radiographic (mandibular MBL) parameters in RA (with and without PD), PD and control groups.

	RA without PD (n = 19)	RA with PD (n = 19)	PD (n = 38)	Controls (n = 14)
**BOP%**	42.6 ± 31‡	37 ± 32	76.2 ± 27*‡§	27.2 ± 29.4
**PPD 3- <5mm**	55.5 ± 16.2§	43.5 ± 17.9**	49.2 ± 17.4§	26.9 ± 14.1*
**PPD≥ 5mm**	0.32 ± 0.74‡	18.2 ± 15.2§	33.4 ± 14.1*‡§	0.4 ± 0.5
**Number of Teeth**	26.8 ± 2.9	25.5 ± 4.1	24.8 ± 4.3	26.1 ± 4.7
**Mandibular MBL mm**	2.9 ± 1	3.8 ± 2.2	5.4 ± 2.6*‡§	3.3 ± 0.7

Differences in the means ± SD of clinical parameters were tested using one-way ANOVA. For multiple comparisons, post-hoc Tukey HSD test was performed.

* compared to RA without PD. P<0.01

‡ compared RA with PD. P<0.01

§ compared to healthy controls. P<0.01

** compared to healthy controls. P<0.05

BOP = Bleeding on probing, PPD = Probing pocket depth, MBL = marginal bone loss

RA = rheumatoid arthritis PD = periodontal disease

### Correlation of cytokines with clinical periodontal parameters

The percentage of BOP sites, number of PPD for 3-<5mm and ≥ 5mm, and number of teeth were the main clinical parameters used to identify associations with cytokine levels using Spearman rank. In order to avoid positive or negative confounding, RA subjects with PD were excluded from the main analysis since they exhibit both disease states. Thus, an analyses was carried out for a singular cohort of 69 subjects after exclusion of 19 subjects with both RA and PD (Tables 5 and 6).

**Table 5:** Correlation of cytokines with clinical parameters* using Spearman rank correlation. The data is presented for biomarkers with adjusted p-values ≤ 0.05 considered significant after using the Benjamini and Hochberg procedure for multiple testing (n = 69).

BOP				PPD 3 - <5mm				PPD ≥ 5mm			
*Analyte*	*r*	*p-value*	*Adjusted p-value*	*Analyte*	*r*	*p-value*	*Adjusted p-value*	*Analyte*	*r*	*p-value*	*Adjusted p-value*
DNER	-0.45	0.000	0.005	CCL25	-0.41	0.000	0.001	DNER	-0.68	1.5E-10	0.003
CD40	-0.42	0.000	0.010	TNFB	-0.41	0.000	0.003	TWEAK	-0.63	5.6E-09	0.006
ST1A1	0.41	0.000	0.015	CCL28	-0.41	0.001	0.004	EN-RAGE	-0.59	7.3E-08	0.008
FGF-19	0.39	0.001	0.020	CXCL5	-0.39	0.001	0.006	ST1A1	0.59	9.2E-08	0.011
EN-RAGE	-0.38	0.001	0.025	LAP TGF-beta-1	-0.39	0.001	0.007	CX3CL1	-0.57	3.4E-07	0.014
NT-3	0.38	0.001	0.030	CCL19	-0.38	0.001	0.009	FGF-19	0.55	1.0E-06	0.017
CASP-8	0.34	0.004	0.035	ADA	-0.38	0.001	0.010	TNFRSF9	-0.48	3.2E-05	0.019
TWEAK	-0.32	0.007	0.040	SCF	-0.36	0.002	0.012	CCL8	-0.40	7.7E-04	0.022
CD244	0.28	0.018	0.045	CXCL11	-0.36	0.003	0.013	NT-3	0.38	1.3E-03	0.025
CX3CL1	-0.25	0.036	0.050	CD40	0.35	0.003	0.015	STAMPB	-0.37	1.9E-03	0.028
				SIRT2	-0.35	0.003	0.016	LIF-R	0.33	5.8E-03	0.031
				CCL11	-0.35	0.003	0.018	CCL25	0.33	6.1E-03	0.033
				CASP-8	-0.34	0.004	0.019	CD5	0.31	9.4E-03	0.036
				IL-12B	-0.33	0.006	0.021	CASP-8	0.31	9.7E-03	0.039
				IL-10	-0.32	0.007	0.022	CD6	0.29	1.5E-02	0.042
				SLAMF1	-0.31	0.009	0.024	SIRT2	-0.28	1.8E-02	0.044
				TRANCE	-0.31	0.009	0.025	CCL20	-0.26	3.3E-02	0.047
				CXCL1	-0.31	0.009	0.026	CD40	-0.25	3.6E-02	0.050
				CXCL6	-0.31	0.009	0.028				
				CCL4	-0.30	0.011	0.029				
				TGFA	-0.30	0.011	0.031				
				CD244	-0.29	0.014	0.032				
				TRAIL	-0.29	0.015	0.034				
				IL-10RB	-0.28	0.018	0.035				
				IL-7	-0.28	0.019	0.037				
				CD6	-0.28	0.019	0.038				
				MMP-10	-0.27	0.023	0.040				
				MMP-1	-0.27	0.024	0.041				
				CCL7	-0.27	0.027	0.043				
				CCL3	-0.26	0.028	0.044				
				CD5	-0.26	0.034	0.046				
				Beta-NGF	-0.25	0.040	0.047				
				TNFRSF9	-0.24	0.044	0.049				

*BOP = Bleeding on probing, PPD = Probing pocket depth

**Table 6:** Correlation of cytokines with MBL* in mandibular molars and premolars and number of teeth using Spearman rank correlation. The data is presented for biomarkers with adjusted p-values ≤ 0.05 considered significant after using the Benjamini and Hochberg procedure for multiple testing (n = 69).

MBL				Number of Teeth			
*Analyte*	*r*	*p-value*	*Adjusted p-value*	*Analyte*	*r*	*p-value*	*Adjusted p-value*
TWEAK	-0.52	4.3E-06	2.9E-03	CDCP1	-0.53	2.38E-06	0.002
DNER	-0.51	6.8E-06	5.9E-03	CCL8	0.51	6.59E-06	0.004
CX3CL1	-0.50	1.2E-05	8.8E-03	STAMPB	0.50	1.13E-05	0.006
STAMPB	-0.50	1.3E-05	1.2E-02	CX3CL1	0.50	1.37E-05	0.007
EN-RAGE	-0.46	6.3E-05	1.5E-02	TNFRSF9	0.44	1.84E-04	0.009
FGF-19	0.46	7.2E-05	1.8E-02	DNER	0.40	6.29E-04	0.011
CCL8	-0.45	1.1E-04	2.1E-02	SIRT2	0.38	1.37E-03	0.013
TNFRSF9	-0.45	1.2E-04	2.4E-02	TWEAK	0.36	2.56E-03	0.015
CDCP1	0.37	2.0E-03	2.6E-02	FGF-19	-0.35	2.86E-03	0.017
SIRT2	-0.34	4.2E-03	2.9E-02	EN-RAGE	0.35	3.34E-03	0.019
ST1A1	0.33	5.1E-03	3.2E-02	CXCL10	0.34	3.87E-03	0.020
HGF	0.30	1.1E-02	3.5E-02	CXCL11	0.31	8.54E-03	0.022
NT-3	0.30	1.1E-02	3.8E-02	CCL11	0.31	9.36E-03	0.024
IL-10RA	-0.27	2.7E-02	4.1E-02	VEGF-A	0.30	1.36E-02	0.026
MMP-10	-0.25	3.7E-02	4.4E-02	CCL4	0.30	1.38E-02	0.028
CXCL10	-0.25	4.0E-02	4.7E-02	CCL20	0.28	2.07E-02	0.030
CD6	0.25	4.1E-02	5.0E-02	CXCL5	0.28	2.20E-02	0.031
				CXCL6	0.27	2.28E-02	0.033
				MMP-1	0.27	2.53E-02	0.035
				IL-10	0.27	2.63E-02	0.037
				IL-7	0.27	2.68E-02	0.039
				TGFA	0.26	2.81E-02	0.041
				ST1A1	-0.26	3.43E-02	0.043
				IL-18R1	-0.25	3.45E-02	0.044
				CCL23	0.25	3.82E-02	0.046
				HGF	-0.24	4.34E-02	0.048
				LAP TGF-beta-1	0.24	4.82E-02	0.050

MBL = marginal bone loss

For BOP, five out of ten cytokines were positively correlated (Table 5). The correlation between number of shallow pockets of 3-<5mm was significant for 33 cytokines. All cytokines, except for CD40, correlated negatively with the number of shallow pockets (Table 5). Concerning number of deep pockets (≥5mm), a correlation was significant for a total of 18 cytokines out of which ten were negatively correlated and eight were positively correlated (Table 5). For all Spearman rank correlations between clinical parameters with the 66 different cytokines (66 × 5), refer to S3 Table in supporting information.

### Correlation of cytokines with mandibular MBL and number of teeth

Marginal bone loss in mandibular molars and premolars and number of teeth were correlated against cytokines. These are shown in Table 6. Seventeen cytokines correlated significantly with mandibular MBL. The number of teeth were associated with 27 cytokines out of which five were negative coefficients (Table 6).

### Subgroup analysis for correlation of cytokines in RA subjects

A subgroup analysis was performed for RA subjects to compare which cytokines correlate with clinical variables under the influence of periodontal disease. The results are shown in Tables 7 and 8.

**Table 7:** Correlation of cytokines with BOP, PPD 3-<5 and PPD ≥ 5mm in RA subjects with and without PD using Spearman rank correlation. Adjusted p-values ≤ 0.05 considered significant after using the Benjamini and Hochberg procedure for multiple testing.

RA WITH PD				RA WITHOUT PD			
**BOP**							
*Analyte*	*Spearman R*	*p-value*	*Adjusted p-value*	*Analyte*	*Spearman R*	*p-value*	*Adjusted p-value*
IL-7	-0.67	0.002	0.002	SLAMF1	-0.47	0.044	0.017
IL-10RB	-0.61	0.006	0.007	ADA	-0.58	0.009	0.050
CXCL10	-0.60	0.006	0.009				
uPA	-0.60	0.006	0.011				
MMP-10	-0.60	0.006	0.013				
TGF-beta-1	-0.60	0.007	0.015				
TWEAK	-0.59	0.008	0.017				
CX3CL1	-0.57	0.011	0.020				
Flt3L	0.56	0.012	0.022				
CXCL6	-0.56	0.012	0.024				
EN-RAGE	0.56	0.013	0.026				
CCL25	-0.56	0.013	0.028				
CXCL5	-0.56	0.013	0.030				
SCF	-0.55	0.016	0.033				
FGF-19	-0.54	0.017	0.035				
CXCL1	-0.52	0.022	0.037				
SLAMF1	-0.51	0.025	0.039				
MMP-1	-0.50	0.029	0.041				
TRAIL	-0.49	0.033	0.043				
CCL19	-0.47	0.041	0.046				
CD244	-0.47	0.041	0.048				
**PPD 3 - <5mm**							
*Analyte*	*Spearman R*	*p-value*	*Adjusted p-value*	*Analyte*	*Spearman R*	*p-value*	*Adjusted p-value*
CCL8	-0.75	0.000	0.001	TGFA	-0.55	0.01	0.03
PD-L1	-0.72	0.000	0.003	MMP-10	-0.48	0.04	0.05
CASP-8	-0.70	0.001	0.004				
CCL13	-0.70	0.001	0.005				
STAMPB	-0.70	0.001	0.007				
CXCL11	-0.70	0.001	0.008				
CD244	-0.69	0.001	0.009				
CX3CL1	-0.68	0.001	0.011				
TNFB	-0.67	0.002	0.012				
IL-15RA	-0.65	0.002	0.014				
CCL2	-0.64	0.003	0.015				
CCL20	-0.63	0.004	0.016				
SLAMF1	-0.62	0.005	0.018				
CCL19	-0.60	0.007	0.019				
Beta-NGF	-0.60	0.007	0.020				
IL-10RB	-0.59	0.008	0.022				
IL-10	-0.58	0.009	0.023				
SIRT2	-0.58	0.009	0.024				
CXCL6	-0.57	0.011	0.026				
4E-BP1	-0.56	0.012	0.027				
IL-10RA	-0.55	0.014	0.028				
IL-12B	-0.55	0.015	0.030				
MIP-1alpha	-0.54	0.018	0.031				
IL-7	-0.53	0.018	0.032				
CXCL10	-0.53	0.020	0.034				
ADA	-0.53	0.020	0.035				
CXCL1	-0.52	0.022	0.036				
CCL23	-0.52	0.024	0.038				
VEGF-A	-0.51	0.024	0.039				
CXCL5	-0.51	0.024	0.041				
CCL4	-0.51	0.027	0.042				
uPA	-0.50	0.030	0.043				
LIF-R	-0.49	0.032	0.045				
CCL25	-0.48	0.037	0.046				
SCF	-0.48	0.038	0.047				
FGF-19	-0.47	0.044	0.049				
NT-3	-0.47	0.044	0.050				
**PPD ≥ 5mm**							
*Analyte*	*Spearman R*	*p-value*	*Adjusted p-value*	*Analyte*	*Spearman R*	*p-value*	*Adjusted p-value*
CXCL10	-0.49	0.034	0.036				
IL-10	-0.46	0.048	0.050				

*BOP = Bleeding on probing, PPD = Probing pocket depth

RA = rheumatoid arthritis PD = periodontal disease

**Table 8:** Correlation of cytokines with mandibular MBL* and number of teeth in RA subjects with and without PD using Spearman rank correlation. Adjusted p-values ≤ 0.05 considered significant after using the Benjamini and Hochberg procedure for multiple testing.

RA WITH PD				RA WITHOUT PD			
**MBL**							
*Analyte*	*Spearman R*	*p-value*	*adjusted p value*	*Analyte*	*Spearman R*	*p-value*	*Adjusted p-value*
CCL7	0.62	0.015	0.050	ADA	0.61	0.007	0.017
				CST5	0.57	0.013	0.033
				SLAMF1	0.51	0.029	0.050
**Number of Teeth**							
*Analyte*	*Spearman R*	*p-value*	*Adjusted p-value*	*Analyte*	*Spearman R*	*p-value*	*Adjusted p-value*
CCL7	-0.52	0.022	0.025	DNER	0.46	0.049	0.050
CXCL9	-0.51	0.026	0.030				
CD244	-0.50	0.030	0.035				
Flt3L	-0.48	0.040	0.040				
IL-15RA	-0.47	0.044	0.045				
CDCP1	-0.46	0.048	0.050				

*MBL = marginal bone loss

RA = rheumatoid arthritis PD = periodontal disease

## Discussion

Analytical techniques with greater sensitivity can help explore a broader range of periodontal disease-associated biomarkers as compared to conventional enzyme-linked immunosorbent assays, which have been used extensively in research in this field [15]. In our study, we employed the Proximity Extension Assay (PEA) technology which allowed us to analyze up to 92 human proteins related to inflammation in RA, PD and healthy subjects. Both PD and RA exhibit considerable heterogeneity in the nature of the inflammatory response between individuals due to genetic, epigenetic and environmental factors [16]. This may be reflected by a greater dispersion of cytokines within and between groups which is why the aim of this study was to focus on the correlation between inflammatory cytokines and periodontal parameters rather than compare inter-group cytokine levels.

Periodontal disease occurs in susceptible individuals in which disease severity is determined by the host immuno-inflammatory response to a polymicrobial challenge [17]. This is deemed as a defense response and leads to the clinical manifestation of PD. Inflammation in the periodontal connective tissue leads to attachment loss between bone and tooth, with epithelial cells proliferating apically along the root surface, thus leading to the formation of a deep pocket. As the periodontal pocket deepens, so too does the extent of the inflammatory infiltrate [18].

### Novel markers of interest

In our study, by defining PD as three or more pockets ≥ 5mm, we were able to evaluate periodontal disease at a specified diagnostic threshold. We have identified significant correlations for several markers with all clinical parameters. Fibroblast growth factor 19 (FGF-19), neurotrophin 3 (NT-3) and sulfotransferase 1A1 (ST1A1) were positively correlated with BOP, deep periodontal pockets (PPD measuring ≥5mm) and MBL. This is interesting in light of the fact that both soft and hard tissue parameters are associated with similar systemic inflammatory proteins. FGF-19 is an endocrine FGF that can be released into the bloodstream to act throughout the body. It is a late-acting hormone which can induce the secretion of insulin and glucagon to regulate glucose homeostasis [19]. NT-3 is expressed by human periodontal ligament cells and can induce mRNA expression of bone related proteins in these cells with the implication that it may have bone regenerative potential [20, 21]. Human ST1A1 belongs to an enzyme family, predominantly found in the liver that regulates the activities of drugs, endogenous metabolites and xenobiotics [22]. No data is available regarding its role in either RA or PD. Our findings are the first to report these novel markers and their serological association with periodontal conditions.

Our results showed a consistent pattern for parameters (BOP, PPD≥ 5mm and MBL) that were positively correlated with FGF-19 but inversely correlated to EN-RAGE, DNER, TWEAK, and CX3CL1. These proteins are abundantly expressed by immune cells including granulocytes, monocytes, macrophages as well as fibroblasts and neuronal cells (DNER). They are generally recruiters and/or stimulators of other inflammatory mediators, or part of a cellular network which influences outcomes of immunological significance. Furthermore, they are known to be expressed locally in synovial and gingival tissues [23–26]. The inverse correlation for periodontal parameters with some markers could be due to a profound yet localised inflammatory milieu with a higher concentration being consumed or engaged with receptor sites in the local tissues. This is also evident from our results pertaining to the number of teeth as most markers which positively correlated with parameters of disease such as BOP, deep pockets and MBL were inversely related with number of teeth and vice versa.

### T cell markers

The CD5 and CD6 receptors are special in the way of being the only two lymphocytic receptors that can express both cell-surface and secreted proteins which belong to the scavenger receptor cysteine-rich superfamily (SRCR-SF). Functionally, they are associated to the antigen-specific receptor complex present on T (TCR/CD3) and B (BCR) cells, where they engage in the modulation of the activation and differentiation signals delivered by that receptor complex [27]. The CD5 and CD6 receptors, both exist as membrane bound and as soluble receptors circulating in serum. CD5 also plays a role in inhibiting peripheral blood T-cell signaling [28]. Since it is an important regulator of T-cell immune responses, CD5 regulation corresponds to one aspect of immune homeostasis [29]. Any breach in this tightly regulated mechanism leads to inflammation mediated by T cells which forms the basis of autoimmune pathology [30]. In recent studies, CD6 has been identified as a susceptibility gene in both multiple sclerosis and RA [31, 32]. Our study showed that both CD5 and CD6 levels were associated with increasing number of deep pockets with CD6 also correlating with MBL. Previous studies have shown increased levels of CD5 in peripheral lymphocytes of patients with PD [33]. Even though the associations are weak, they maintain that systemic T- cell aberration is an influence on periodontal disease pathogenesis.

### Apoptotic markers

Proteins related to apoptosis (programmed cell death) were found to be associated positively with BOP (CASP-8), deep pockets (CASP-8, HGF) and MBL (CDCP1). Caspases are not only involved in apoptosis but also in the induction of inflammation. In fact, there is enough supporting evidence that both processes are linked at various levels [34]. Caspase-8 has been shown to have additional functions unrelated to cell death, including T cell activation, cell motility, and tumor metastasis [35]. Its activity in PD has been demonstrated through sensitization of osteoblasts to apoptotic signals and that the apoptotic process is associated with an early activation of caspase-8 and 3 in these osteoblasts [36]. HGF (hepatocyte growth factor) is a cytokine with pleiotropic properties produced by mesenchymal cells including oral fibroblasts. Elevated HGF concentrations have a convincing role as a marker of chronic inflammation including periodontal disease as patients with severe PD have higher HGF concentrations in gingival crevicular fluid (GCF), saliva, and serum [37]. Both pro- and anti-apoptotic effects of HGF have been reported depending on the cell type and state as well as on the signal transducers with the involvement of caspase-8 [38]. CDCP1 is a glycoprotein which is reportedly expressed in normal epithelial cells, and overexpressed in epithelial tumor cells such as colon, breast, lung, renal and pancreatic cancers and is said to regulate mechanisms related to inhibition of cell death [39, 40].

### IL-10 and chemokines

Interleukin 10 (IL-10) is a potent anti-inflammatory cytokine that plays an essential, role in preventing inflammatory and autoimmune diseases. IL-10 RA and IL-10 RB are receptors required for IL10-induced signal transduction. Suppression of IL-10 expression can enhance inflammatory response to microbial challenge but also increase the risk of developing a number of autoimmune diseases [41]. Our analysis revealed an inverse correlation with both IL-10 and its receptors for most of the periodontal parameters, especially in the RA group. Chemokines were also found to be inversely associated with the number of pockets and MBL except for CCL25, the only chemokine which associated positively with deep pockets. Chemokine expression in synovial tissue, fluid and sera are known to be increased in RA subjects [42]. However, suppression of chemokines can be due to non-steroidal anti-inflammatory drugs, corticosteroids and traditional disease-modifying anti-rheumatic drugs (DMARD) which are known to exert multiple anti-inflammatory properties including chemokine inhibition [43]. In periodontal disease, the chemokine expression in local tissues may be suppressed due to mechanisms related to microbial challenge. Low levels of chemokines in local tissues may therefore be lower in the circulatory system, hence, lower levels are detected in the sera of PD subjects [44, 45].

### Systemic burden of periodontal disease

Given the number of possible correlations, the highest number of significant correlations was seen in the cohort comprising of 69 subjects (105 correlations). This was followed by the RA with PD group having over half the number of significant correlations (68 correlations). The least number of correlations was found in the RA without PD group which reported correlations almost nine times less than RA with PD subjects (8 correlations).

It is notable how measures of periodontal inflammation are positively associated with novel proteins whose functions and relationship to either PD or RA is not completely known at the moment but offers a new direction and scope for future research. Also, our results have highlighted the importance of aberrant T-cell function, apoptotic processes and a deficiency of anti-inflammatory mechanisms as important biological processes related to both periodontal disease and rheumatoid arthritis.

The limitations in our study pertain to the small sample size being recruited at a single site. A larger sample size would have allowed a better representation of study groups, allowing results to be generalized with ease.

## Conclusion

Our results demonstrate an association between bleeding on probing, deep periodontal pockets and marginal bone loss with systemic immuno-inflammatory markers. Shallow periodontal pocketing does not impart a systemic inflammatory burden. Apoptotic cell death markers may be important determinants of disease severity.

## Supporting information

S1 File Questionnaire. (PDF)

S1 Table Data on 66 assayed proteins for all subjects*. NAN = Not a number. NAN data cells were treated as zero in all calculations.
(PDF)

S2 Table Data on BOP, PPD 3-<5mm, PPD ≥5mm and mandibular MBL for all subjects*. NA = Not available. NA data cells were treated as zero in all calculations.
*R = Rheumatoid arthritis (green), P = Periodontal disease (orange), H = Healthy (grey).
(PDF)

S3 Table Data for all Spearman rank correlations between clinical parameters and 66 cytokines for study cohort (n = 69). Significant results (p-value < 0.05) are in blue while correlations ≥ 0.05 are shown in black.
(PDF)
