# Supplementary material for: Correlation of serum cytokines, chemokines, growth factors and enzymes with periodontal disease parameters
Source: PLoS One. 2017 Nov 30;12(11):e0188945. doi: 10.1371/journal.pone.0188945 (PMC5708747; doi:10.1371/journal.pone.0188945)
Supplement: S1 File — (PDF) [file pone.0188945.s001.pdf]

PROTOCOL 1: Questionnaire

Date: _______________

Patient Serial Number: _____________

**Name:**	**Marital Status:**○ **Single** ○ **Married**○ **Divorced Widow /er** ○
**Telephone:**	**How many children?**
**Address:**	**Salary per month:**
**Date of Birth: (dd/mm/yy)**	**Occupation:**
**Age:**	**Education:**
**Gender:**	

INCLUSIVE CRITERIA:

	**Yes**	**No**
Are you a Pakistani resident?	☐	☐
Written information	☐	☐

EXCLUSION CRITERIA:

	**Yes**	**No**
Do you or did you have Hepatitis/HIV/AIDS?	☐	☐
Do you suffer from Gout or Osteoarthritis?	☐	☐
Do you use any narcotic drugs?	☐	☐
Antibiotic treatment during last 3 months?	☐	☐

MEDICAL HISTORY:

Are you receiving any medical treatment: Yes/No

If yes, please refer to the medication protocol.

Are you pregnant?

Were you ever admitted to a hospital for operation? If yes, when and for what?

Do you have Rheumatoid arthritis (RA)? : Yes/No

Do you have family history of RA? Yes/No

When were you diagnosed for RA? At what age did the symptoms start?

What medication do you use for RA; which one, what dosage and when did you start the medication? (Refer to medication chart on pg 4)

Do you have diabetes mellitus? : Yes/No

Do you have a family history of diabetes? : Yes/No

When were you diagnosed with diabetes?

Is it IDDM /Type 1? Yes/No

Is it NIDDM/Type 2? Yes/No

Do you have hypertension? CVD? Yes/No

Do you have renal disorders? Yes/No

Do you have Sjögren’s syndrome? Yes/No

History of Infectious disease(s)?

- Cholera
- Typhoid Fever
- Tuberculosis
- Malaria
- Dengue

Do you have any other medical disease: Yes/No

If yes, please specify: ____

NAME OF MEDICATION	DOSAGE<br><br>(mg, units, puffs, drops)	PURPOSE<br><br>Why do you take it?	DURATION<br><br>Start date of medication

HABITS:

Cigarette smoking: Yes / No

Since when? ______________________

How many per day? __________________

Betel nut chewing: Yes / No and since when?

How many per day? __________________

Tobacco chewing: Yes / No and since when?

How many per day? __________________

Snuff: Yes / No

How many per day? ___________________

Do you use (1) Pan with betel nut: Yes/No (2) Gutkha: Yes/No (3) Only Betel nut: Yes/No
(4) Pan without betel nut: Yes/No

How many per day? ____

ORAL HEALTH MAINTENANCE

Brushing teeth: Yes / No

How many times do you brush teeth? Please specify: ______________________________

Use of Meswak: Yes / No

Do you use any other means to maintain oral hygiene? Yes / No

If yes, please specify: ____

Which mouthwash do you use? Please specify: ___________________________

ORAL HEALTH STATUS

Number of teeth present: ____________

Bleeding gums: Yes / No

Swollen gums: Yes/ No

Painful gums: Yes / No

Pain in teeth: Yes / No

Pain on chewing: Yes / No

Mobile teeth? Yes/No

Oral ulcers? Yes /No

Do you have sensitive teeth? Yes / No

Do you have pain on eating sweets? Yes/ No

How often do you have pain in teeth? Please specify: ___________________________________

How often do your gums bleed? Please specify: _______________________________________

How often do you develop ulcers in your mouth?

Please specify: ____

Do you have any dryness of mouth? Yes/ No

Temporomandibular Joint (TMJ)

Problems in TMJ? ____________________________________

Opening? ____________________________________

Pain? ____________________________________

DENTAL VISITS

Have you ever visited a dentist? Yes / No

If yes, when did you last visit the dentist? Please specify: ______________________________

How often do you visit a dentist? Please specify____________________________________

What was the treatment you underwent in your last dental visit? __________________________

Do you like going to dentist? Yes / No
