# Supplementary material for: Correlation of serum cytokines, chemokines, growth factors and enzymes with periodontal disease parameters
Source: PLoS One. 2017 Nov 30;12(11):e0188945. doi: 10.1371/journal.pone.0188945 (PMC5708747; doi:10.1371/journal.pone.0188945)
Supplement: S1 Table — NAN = Not a number. NAN data cells were treated as zero in all calculations. (PDF) [file pone.0188945.s002.pdf]

#LOD-Max	2.12	1.06	1.45	0.28	1.33	0.87	0.96	0.85
Subject ID	101_IL-8	102_VEGF-A	105_MCP-3	107_CDCP1	108_CD244	109_IL-7	110_OPG	111_LAP TGF-beta-1
R1	10.27	8.74	NAN	3.72	5.81	2.26	10.11	4.69
R2	11.98	9.70	NAN	2.90	4.87	1.32	9.77	3.85
R3	11.92	8.42	NAN	2.72	5.05	1.04	9.66	4.55
R4	8.38	9.47	2.56	5.05	6.84	3.25	11.12	5.02
R5	10.58	9.89	NAN	4.17	5.02	1.49	10.65	5.25
R6	6.50	8.38	2.29	3.07	5.45	1.46	10.39	3.84
R7	9.61	9.69	NAN	3.03	6.22	4.07	10.13	5.13
R8	12.05	10.98	2.09	3.45	6.10	2.68	10.37	5.00
R9	7.66	9.79	NAN	3.95	6.17	2.82	10.39	4.54
R10	13.40	10.69	NAN	2.97	5.57	2.05	9.74	4.58
R11	12.90	10.70	2.29	2.92	6.96	3.91	10.19	6.05
R12	8.04	10.22	NAN	3.62	4.32	1.53	9.57	3.30
R13	13.91	8.42	3.44	4.00	5.85	2.97	10.67	4.52
R14	14.66	9.59	2.62	3.02	6.22	4.48	10.15	4.29
R15	10.67	9.31	NAN	2.83	4.33	1.48	9.10	3.79
R16	9.87	9.82	NAN	4.59	4.30	0.88	10.89	4.83
R17	8.91	10.92	4.26	3.08	6.38	6.80	10.02	6.97
R18	10.72	10.67	3.28	2.99	6.24	5.86	9.92	7.36
R19	9.41	10.32	NAN	2.78	6.13	3.83	10.22	4.81
R20	9.28	9.74	NAN	4.91	5.78	1.70	10.30	4.19
R21	9.73	10.02	NAN	4.53	5.75	1.70	10.96	3.88
R22	14.78	9.15	4.74	4.58	6.35	2.66	10.71	4.86
R23	11.41	10.48	NAN	3.84	6.04	1.58	10.67	4.44
R24	13.48	9.22	1.50	3.47	4.08	1.03	9.79	3.51
R25	14.54	11.59	6.84	4.82	6.12	4.84	10.27	6.39
R26	14.82	11.50	9.22	5.52	7.09	4.54	11.21	6.35
R27	10.43	10.33	3.81	4.28	7.07	5.14	10.29	6.69
R28	12.84	11.50	5.02	3.74	6.22	5.46	9.71	7.86
R29	9.50	12.01	5.10	5.07	6.88	6.03	10.28	7.19
R30	9.08	11.15	5.52	5.55	7.22	5.38	11.48	7.26
R31	10.10	11.10	3.78	4.49	6.71	5.04	10.47	7.29
R32	10.33	11.57	4.72	5.56	6.68	5.25	10.80	7.32
R33	9.98	12.40	2.97	2.78	6.80	5.52	10.13	7.38
R34	9.34	11.84	2.39	4.47	7.03	6.11	11.72	7.70
R35	12.10	10.55	2.89	4.44	6.80	4.84	10.29	5.51
R36	13.50	10.43	2.64	5.15	6.51	1.92	10.34	4.71
R37	7.89	10.18	NAN	3.91	6.54	3.42	10.59	5.67
R38	11.39	10.00	4.08	4.10	6.32	5.24	10.24	6.80
P1	7.54	9.33	NAN	5.69	5.24	1.35	9.97	4.95
P2	11.50	10.11	3.11	5.11	7.54	4.59	10.38	5.07
P3	11.69	9.69	1.55	5.38	5.67	2.25	9.90	4.52
P4	13.08	9.68	NAN	3.53	6.11	3.46	10.05	4.86
P5	10.82	9.28	NAN	3.83	5.47	1.19	10.72	4.07
P6	12.43	9.00	3.20	2.92	6.69	4.73	10.12	5.64
P7	13.38	9.00	1.68	5.74	6.44	2.35	10.69	4.58
P8	6.38	8.87	NAN	3.18	5.98	2.49	9.16	4.30
P9	12.26	10.34	NAN	3.27	6.70	4.56	10.61	5.14
P10	10.71	10.08	1.95	3.21	6.59	4.60	10.41	5.56

P11	11.66	9.79	NAN	3.02	5.87	2.96	10.28	4.91
P12	13.27	10.15	4.81	3.51	6.79	4.67	10.06	6.66
P13	10.82	9.87	NAN	4.74	6.21	2.51	10.65	4.64
P14	14.13	10.15	2.91	3.69	6.52	3.08	9.58	5.38
P15	8.31	8.89	1.79	4.17	6.30	3.86	9.73	4.77
P16	14.22	10.68	NAN	3.21	6.10	2.55	11.39	4.57
P17	10.01	10.26	2.74	3.45	6.53	4.75	9.93	5.32
P18	14.75	10.54	10.69	4.61	6.99	4.54	9.88	6.14
P19	11.74	10.47	2.22	3.17	6.73	4.14	10.40	5.30
P20	14.75	9.36	1.71	3.36	6.59	3.77	9.62	5.17
P21	7.59	9.30	NAN	3.27	6.56	3.94	9.40	4.82
P22	14.18	10.32	4.67	2.71	6.31	3.64	10.05	4.78
P23	14.95	10.40	6.37	3.49	7.07	5.18	10.44	5.19
P24	14.17	10.49	4.24	4.24	6.39	3.86	9.74	6.79
P25	14.87	9.89	5.25	2.54	6.82	4.44	9.72	5.90
P26	15.09	10.50	7.14	3.30	6.89	4.51	10.11	5.71
P27	14.73	10.46	1.77	3.99	5.48	1.44	9.70	4.16
P28	7.51	9.48	1.67	3.28	5.95	1.85	10.06	4.64
P29	8.37	8.25	NAN	4.69	6.10	1.73	10.56	4.46
P30	9.34	9.56	1.74	4.06	6.11	5.27	10.05	5.51
P31	14.72	10.40	2.33	4.13	5.22	2.60	10.07	4.75
P32	14.51	9.83	2.49	3.74	4.34	1.18	10.10	3.73
P33	14.35	9.96	5.89	3.78	6.46	5.16	10.20	5.39
P34	11.27	10.19	3.12	3.49	6.31	4.40	9.37	6.00
P35	14.69	11.80	11.71	4.40	6.99	3.18	9.93	5.53
P36	14.90	11.28	8.97	3.75	7.90	4.68	10.38	6.99
P37	14.72	9.99	2.12	3.75	5.65	1.72	9.96	4.16
P38	14.46	11.05	10.48	3.58	6.83	4.91	10.69	5.06
H1	12.96	9.73	1.49	2.81	5.40	1.26	9.58	4.26
H2	14.83	9.51	5.77	4.64	6.60	3.93	10.70	5.06
H3	13.15	11.43	4.49	3.50	6.65	5.33	9.95	7.12
H4	13.20	10.35	5.61	3.33	6.79	5.44	9.60	7.83
H5	13.30	10.93	4.19	3.75	6.46	5.15	9.80	7.35
H6	12.13	11.05	3.45	3.15	7.05	5.52	9.90	8.04
H7	13.46	10.20	4.74	2.77	6.88	4.56	9.81	7.25
H8	13.14	10.64	4.85	2.46	6.57	4.96	9.85	7.20
H9	12.43	11.42	4.80	3.93	7.08	5.39	9.83	7.65
H10	12.95	10.12	4.10	2.68	6.39	5.30	9.51	7.60
H11	13.62	11.19	6.41	3.09	6.50	5.15	9.76	7.27
H12	12.98	10.90	4.53	3.13	7.16	5.75	10.15	8.09

0.88	0.89	0.28	1.44	0.42	1.32	0.37	-0.22	0.89
112_uPA	113_IL-6	115_MCP-1	117_CXCL11	120_TRAIL	122_CXCL9	123_CST5	126_OSM	128_CXCL1
10.00	3.95	9.13	2.61	6.57	8.88	6.06	4.78	3.09
9.59	3.20	6.91	NAN	5.66	4.27	5.26	5.61	2.80
9.76	4.47	7.46	NAN	6.41	4.77	6.17	4.40	2.02
10.69	5.16	9.96	2.55	7.34	9.13	7.61	4.93	6.73
9.14	3.99	0.63	4.20	4.99	7.43	5.62	5.44	2.36
9.62	2.07	8.32	NAN	5.00	5.50	5.44	2.95	0.97
10.41	4.54	8.84	NAN	6.57	7.21	6.02	5.76	4.97
10.17	4.11	8.99	2.90	5.53	6.61	6.46	5.35	4.94
10.12	4.32	9.04	1.87	8.26	11.24	6.48	4.71	5.40
9.45	4.33	9.64	1.77	6.42	7.03	6.08	6.01	2.97
10.61	5.40	8.79	2.07	6.58	7.70	6.25	5.58	6.30
9.59	2.95	5.99	NAN	6.18	3.32	4.95	4.44	2.29
9.71	5.74	10.71	2.50	5.74	8.71	5.46	6.10	6.45
9.99	6.53	9.94	1.47	5.63	6.99	5.73	7.11	4.53
8.85	2.07	6.41	NAN	4.87	4.94	6.44	4.63	2.36
9.32	3.14	6.11	NAN	5.59	4.19	5.86	4.65	2.16
9.86	4.04	10.33	9.36	7.71	9.10	6.39	4.64	8.99
10.23	4.34	10.75	8.19	8.00	7.89	7.23	5.47	9.61
9.98	3.46	8.19	1.52	6.52	8.55	6.24	4.55	5.27
10.13	4.56	7.85	NAN	6.94	7.34	6.45	4.54	2.63
10.31	4.38	8.36	NAN	6.47	6.36	7.74	4.83	2.75
10.44	9.35	11.87	1.68	7.19	8.73	6.59	7.51	8.45
10.10	5.05	9.79	1.59	6.53	7.63	6.72	4.65	2.38
8.81	4.17	5.92	NAN	5.71	3.15	4.73	4.63	2.38
10.37	7.53	11.36	8.37	7.07	8.41	5.81	7.31	9.42
11.18	7.93	12.24	4.35	6.98	8.92	7.22	8.17	8.60
10.55	3.96	10.42	8.48	7.38	9.20	8.37	5.45	8.13
10.44	7.03	11.21	6.86	8.09	8.26	7.45	7.14	9.60
10.44	7.18	9.66	8.14	7.58	9.11	8.15	4.15	9.51
11.09	6.46	10.99	9.58	7.89	10.45	7.31	2.26	9.72
10.62	5.32	9.91	7.44	7.79	9.03	7.10	5.79	9.89
10.68	5.03	10.64	8.55	8.21	9.86	6.70	5.70	9.17
10.05	5.37	10.24	8.43	7.50	8.87	6.98	5.02	10.25
10.83	9.55	11.57	7.61	7.03	8.26	7.15	7.96	10.58
10.73	4.99	10.27	4.22	6.68	7.83	6.74	6.07	8.60
10.16	7.50	12.44	2.66	6.29	9.65	6.39	5.53	2.90
10.69	5.72	3.17	2.21	7.80	9.26	6.95	5.74	7.23
10.41	3.67	9.95	7.46	6.98	7.10	6.64	5.35	9.04
9.34	2.91	7.88	NAN	4.72	3.11	6.28	4.51	1.93
10.55	4.42	9.41	1.91	7.39	7.57	6.34	6.06	5.34
9.77	4.07	7.84	NAN	5.25	6.16	5.88	4.77	6.26
10.04	4.77	8.93	1.45	7.02	7.45	5.94	6.34	5.81
9.61	3.38	7.81	NAN	6.37	5.62	5.80	4.52	3.25
10.10	6.33	9.69	2.27	7.40	7.92	6.29	5.54	7.69
10.10	5.98	9.42	2.13	5.95	8.11	6.53	6.09	6.56
9.56	2.94	8.06	NAN	6.40	6.51	6.64	3.90	5.51
10.52	4.94	8.23	2.37	7.02	7.66	6.60	5.00	9.72
10.62	4.71	9.09	5.01	6.64	7.88	7.44	5.86	8.83

10.15	3.39	9.19	1.60	7.09	6.95	6.13	4.84	2.73
10.60	7.21	9.72	7.27	7.51	8.67	6.49	6.17	8.27
10.03	3.86	8.33	NAN	6.86	6.43	6.10	5.35	3.39
10.16	4.82	8.87	1.86	6.73	7.78	6.33	6.59	6.61
10.13	3.45	9.98	1.99	6.42	7.84	6.21	3.93	6.06
9.98	7.38	9.78	NAN	6.41	7.41	6.78	5.49	3.58
10.12	4.29	9.90	5.01	7.16	9.93	6.41	5.60	8.68
10.04	10.20	12.91	2.31	7.18	7.10	5.54	8.44	7.67
9.58	4.30	8.45	3.10	6.51	8.34	5.92	5.49	5.62
10.10	6.73	9.58	2.52	7.22	9.20	6.34	6.94	9.63
10.07	3.47	8.57	NAN	6.54	6.94	6.87	4.39	6.45
10.28	4.08	9.03	1.73	6.77	7.24	6.26	6.73	6.83
10.83	8.65	9.66	3.09	7.36	7.36	6.37	7.14	7.19
10.59	6.17	10.26	7.58	7.43	8.47	6.77	7.13	9.19
10.72	8.99	9.65	3.74	6.68	7.13	6.45	7.17	8.29
10.70	8.16	10.47	7.22	6.95	7.71	6.73	8.20	9.57
9.90	7.65	9.80	2.70	7.08	9.92	6.16	7.26	2.91
9.66	2.93	8.32	NAN	6.38	6.61	6.39	2.93	2.64
9.57	3.51	6.94	NAN	6.30	7.09	6.66	4.46	4.28
10.22	5.41	9.60	2.12	6.80	8.91	6.65	4.80	7.01
10.28	5.20	8.36	NAN	7.50	5.35	6.09	7.33	4.30
9.70	5.42	8.42	1.67	6.65	4.49	5.58	6.15	3.57
10.12	7.93	9.63	3.67	6.59	7.56	6.25	6.00	8.74
9.36	2.49	8.92	1.63	6.19	6.81	6.59	5.05	2.35
10.96	11.12	12.89	6.31	6.25	8.75	8.90	8.29	10.53
10.92	7.32	10.48	4.87	8.19	9.12	6.57	7.97	8.32
10.20	7.21	10.07	NAN	6.16	6.21	6.57	7.63	6.75
10.51	12.86	12.59	1.83	7.23	7.26	8.72	8.38	10.37
9.22	3.51	7.66	2.50	6.55	5.45	5.43	5.90	2.55
9.97	6.87	10.85	1.69	6.22	7.32	7.50	6.89	5.51
10.21	6.40	10.14	7.66	7.57	7.80	6.99	6.89	9.58
10.26	5.36	10.87	7.04	7.62	8.00	6.09	6.30	9.39
10.30	6.99	10.75	7.51	7.58	8.20	6.85	6.61	9.73
10.47	5.52	10.31	7.94	8.64	7.77	7.12	6.74	11.02
10.37	4.20	9.82	7.96	8.05	7.51	6.63	6.75	9.40
10.32	3.69	10.12	6.98	8.07	8.83	7.61	6.57	8.97
10.83	4.22	10.11	7.83	8.60	8.57	7.17	6.54	10.24
10.22	4.10	10.47	7.78	8.03	7.60	6.71	7.48	8.72
10.43	4.39	10.39	7.27	8.37	7.58	7.23	6.99	9.60
10.55	4.53	9.88	7.09	7.93	7.64	7.64	5.74	9.53

-0.13	-0.28	0.62	-0.19	1.47	-1.65	-0.20	1.21	-0.09
130_CCL4	131_CD6	132_SCF	133_IL-18	134_SLAMF1	135_TGFA	136_MCP-4	137_CCL11	138_TNFSF14
5.08	3.49	3.59	7.60	NAN	3.38	1.11	NAN	5.82
1.72	3.67	2.69	6.13	NAN	3.62	NAN	NAN	5.16
2.13	2.89	2.74	5.72	1.77	2.48	NAN	3.19	4.65
5.46	4.56	5.21	7.51	4.19	2.82	3.39	2.21	5.35
3.33	3.44	2.33	5.80	1.59	1.88	NAN	NAN	5.33
0.94	2.29	1.31	6.82	NAN	1.62	NAN	NAN	4.85
3.28	3.08	3.68	6.41	2.12	3.50	1.36	NAN	5.86
4.64	3.73	3.53	6.37	2.04	3.00	0.95	NAN	5.62
4.48	4.40	2.53	7.82	3.42	2.84	2.25	NAN	5.66
1.70	3.16	3.58	7.20	NAN	1.45	0.66	NAN	5.80
7.04	4.58	4.32	7.02	2.90	2.97	1.17	NAN	5.74
0.77	3.06	NAN	5.98	1.51	3.05	NAN	NAN	5.12
5.32	3.70	4.33	9.24	2.01	2.20	0.54	NAN	5.05
7.05	4.05	3.86	6.47	2.01	3.09	0.38	NAN	5.49
1.78	3.21	2.44	5.96	1.67	2.30	NAN	NAN	4.96
1.62	2.55	3.22	7.48	2.46	3.24	NAN	NAN	4.75
7.68	3.78	9.13	7.62	3.47	3.28	3.79	7.89	5.97
7.56	3.15	8.88	7.65	3.30	3.55	2.63	8.10	6.03
3.91	3.51	2.92	6.90	2.08	2.84	1.60	NAN	5.03
3.84	4.07	1.64	7.62	2.01	2.44	0.01	NAN	5.41
4.03	4.23	2.57	6.68	1.75	2.44	0.00	NAN	5.76
7.88	4.22	3.79	7.08	2.73	2.44	0.61	NAN	5.31
4.92	3.45	3.34	7.54	1.49	2.71	0.73	NAN	5.72
2.75	3.21	2.58	6.12	NAN	1.99	NAN	1.22	5.04
8.55	3.69	7.11	7.86	3.69	3.90	4.88	5.50	7.42
7.53	4.46	6.72	8.20	4.23	4.33	3.23	4.57	6.90
7.55	4.91	8.59	7.12	2.98	3.93	4.75	6.19	6.45
8.99	3.92	9.08	7.60	2.89	5.19	3.38	8.51	7.31
5.97	4.33	8.97	8.34	3.81	2.12	4.14	7.77	5.65
7.80	5.15	8.33	8.59	4.59	2.34	4.84	9.03	5.27
7.53	4.62	9.41	9.10	4.10	3.79	4.55	7.37	6.82
7.85	4.02	8.57	8.28	3.79	3.98	4.41	9.32	6.16
7.29	3.54	9.38	7.21	4.22	3.12	4.05	8.24	6.20
7.97	3.98	9.18	8.91	4.31	6.27	3.25	7.76	7.39
4.26	4.34	7.13	7.14	3.29	3.64	4.00	4.65	6.35
6.01	4.22	3.63	7.58	2.87	2.68	0.25	1.42	5.71
4.89	4.81	3.69	7.53	3.58	3.32	-0.07	NAN	6.07
6.51	3.69	7.88	7.64	2.75	3.24	4.18	6.12	6.02
1.70	3.94	2.83	6.37	NAN	-1.33	NAN	NAN	4.81
5.54	4.57	5.38	8.50	3.62	2.92	2.56	2.52	6.32
2.79	3.54	3.22	5.73	NAN	2.01	0.11	NAN	5.39
5.19	3.73	3.71	6.97	1.82	2.64	1.88	NAN	5.71
0.25	3.21	1.60	7.00	2.24	2.35	-0.06	NAN	4.87
7.11	3.63	5.01	6.79	2.33	2.54	3.66	1.84	5.47
2.95	4.57	2.63	8.32	2.69	3.70	0.53	NAN	5.63
-0.06	3.88	3.18	5.90	1.91	0.66	0.32	NAN	4.29
5.41	3.87	3.42	7.84	2.71	3.11	1.43	NAN	5.93
5.39	3.62	5.50	7.15	3.38	3.57	3.84	3.47	6.53

2.21	3.96	2.82	6.38	1.50	2.97	1.80	3.93	6.64
8.52	4.10	7.81	7.72	3.43	3.48	3.48	5.52	6.58
5.47	3.89	2.13	7.21	2.40	3.50	1.37	NAN	5.09
6.30	4.36	3.09	6.54	2.35	3.47	2.71	1.26	6.37
4.07	3.55	5.23	6.79	2.40	2.12	1.70	NAN	4.89
2.57	3.97	3.59	7.10	2.18	3.06	2.87	1.28	6.18
2.97	3.89	6.19	7.05	3.04	3.49	4.52	4.01	6.06
2.67	4.28	4.00	7.03	2.68	2.84	1.72	1.35	6.58
5.65	4.02	5.30	6.78	2.81	3.33	2.02	2.74	5.53
3.09	3.89	3.67	7.11	2.97	3.33	2.58	1.21	6.16
0.51	4.21	3.81	6.13	2.22	1.17	1.37	NAN	5.03
7.22	3.97	6.36	6.69	2.29	3.50	2.62	3.26	5.80
7.92	4.62	5.21	6.36	2.79	4.43	2.53	3.32	6.89
7.27	4.72	9.93	7.59	3.42	3.96	4.36	8.33	7.38
8.80	3.82	6.87	6.64	2.87	3.71	3.32	4.79	6.87
6.86	4.42	7.30	7.29	3.15	3.82	3.60	4.98	7.10
3.81	3.99	2.78	7.26	1.59	3.54	-0.12	NAN	6.19
3.00	4.16	4.03	6.62	2.19	1.50	0.14	NAN	4.42
2.69	3.96	3.77	6.93	2.09	0.93	0.02	NAN	5.32
1.42	3.22	3.56	7.11	2.97	2.45	3.57	1.63	5.48
5.23	3.70	5.13	6.91	2.53	4.38	0.00	NAN	6.23
4.26	3.54	3.97	6.68	2.35	2.25	NAN	NAN	4.58
5.10	3.64	5.14	6.99	2.72	2.79	4.28	3.40	6.42
5.00	3.32	5.53	6.26	2.46	2.39	1.79	2.28	5.03
9.51	5.06	7.26	8.13	4.00	4.40	3.46	4.84	7.53
8.97	5.36	5.81	8.31	3.76	4.99	3.95	4.10	6.94
6.94	3.53	5.17	8.30	2.01	2.05	0.00	NAN	5.98
10.28	3.71	6.56	7.30	3.52	2.91	3.01	3.44	6.26
2.79	3.29	4.36	7.08	2.59	2.23	-0.01	NAN	4.84
6.81	3.58	4.97	6.93	2.49	2.63	1.27	1.87	5.73
8.38	4.28	8.84	7.52	3.66	4.72	2.91	7.45	7.26
7.59	3.55	9.11	7.54	3.74	4.14	3.49	8.10	6.60
7.66	4.02	9.22	8.06	3.10	3.39	3.24	8.26	6.61
8.53	4.28	9.48	8.33	3.50	4.67	3.60	8.25	7.27
7.12	4.08	9.72	7.34	3.36	3.92	2.91	7.58	6.54
6.63	4.12	9.61	7.08	3.47	3.47	3.48	8.65	6.52
7.85	4.87	9.72	8.45	3.21	3.44	3.75	9.03	6.65
9.32	3.54	9.30	7.94	3.54	3.90	2.92	8.41	6.78
8.24	4.14	9.25	7.55	3.57	3.88	3.81	8.50	6.64
7.28	4.39	9.34	7.79	4.00	3.03	3.52	9.23	5.62

0.82	-0.49	1.27	0.99	0.63	1.21	0.71	1.83	0.73
140_IL-10RA	142_MMP-1	143_LIF-R	145_CCL19	148_IL-15RA	149_IL-10RB	151_IL-18R1	152_PD-L1	153_Beta-NGF
1.40	5.70	2.74	2.15	1.68	4.06	8.16	5.03	2.67
1.47	3.70	2.89	NAN	0.95	3.65	7.44	4.46	2.04
NAN	3.92	2.37	NAN	0.91	3.16	6.84	4.03	1.65
0.91	6.02	3.40	2.26	1.32	7.93	8.85	5.98	2.25
NAN	4.33	1.76	NAN	1.78	2.68	9.04	2.78	1.52
1.00	3.89	1.95	NAN	1.33	3.48	7.89	6.01	2.14
NAN	4.34	3.05	NAN	1.05	5.28	8.24	4.53	2.10
NAN	3.73	2.11	2.16	1.61	3.67	8.27	3.43	2.40
1.08	3.48	3.86	NAN	2.06	4.51	8.79	5.86	2.01
0.94	2.59	1.87	1.01	1.32	3.49	7.99	4.53	1.85
NAN	6.35	3.50	1.62	1.73	7.45	8.46	4.86	2.44
NAN	5.72	1.97	NAN	0.70	2.77	7.84	5.43	1.77
NAN	4.95	3.25	NAN	1.30	3.85	8.34	4.31	2.06
1.17	3.35	2.79	NAN	1.13	3.68	8.73	4.68	2.05
NAN	3.19	2.58	NAN	0.64	3.19	7.91	4.34	1.71
1.03	5.51	2.52	NAN	1.42	3.89	7.75	5.17	1.69
NAN	7.35	3.82	6.71	1.53	7.73	8.33	6.12	2.73
1.05	7.09	3.56	6.97	1.73	7.22	8.71	5.44	3.18
NAN	5.14	3.25	1.02	0.87	4.97	7.54	4.58	2.20
1.08	5.47	3.22	NAN	1.23	3.66	8.20	4.53	2.31
1.01	2.58	3.34	1.03	1.14	4.07	8.79	5.56	2.22
1.01	6.96	2.98	1.10	0.90	5.04	8.27	4.26	2.26
0.88	4.33	3.79	2.43	1.26	4.02	8.31	4.99	2.21
0.87	4.17	2.80	1.44	NAN	3.37	7.93	4.31	1.64
1.12	4.86	3.62	4.25	1.90	7.48	8.05	6.16	2.69
0.85	5.02	3.65	2.99	1.90	8.34	9.14	6.19	3.36
1.26	6.08	3.73	6.18	1.56	7.77	8.88	5.69	2.61
NAN	6.90	3.38	8.43	1.53	7.57	7.88	5.39	2.84
0.93	7.32	3.36	9.29	1.99	8.17	8.79	5.72	3.15
1.56	7.07	3.55	11.16	2.23	8.51	9.16	6.09	2.35
1.14	8.21	3.60	10.93	1.92	8.04	8.36	5.68	2.29
1.09	8.12	3.81	8.67	1.63	8.15	8.53	6.01	2.44
1.13	9.69	3.28	8.02	1.31	7.80	7.86	5.78	2.81
1.50	7.28	4.22	10.11	1.59	8.03	8.77	5.79	2.34
1.44	4.34	3.82	4.13	1.23	7.15	7.19	5.99	3.28
1.89	4.23	4.38	5.09	2.04	4.75	9.54	6.10	2.46
1.07	4.56	3.64	2.44	1.48	6.03	8.81	5.79	2.56
1.08	7.46	3.68	4.24	1.65	8.01	8.59	6.03	3.04
NAN	3.56	1.32	NAN	1.79	3.05	8.74	2.38	1.54
NAN	4.41	3.34	2.00	1.39	7.53	9.13	5.11	2.58
NAN	3.47	1.39	NAN	1.91	2.24	8.11	3.29	1.58
0.84	3.32	2.93	NAN	1.23	4.98	8.04	4.34	1.73
0.87	2.43	3.50	NAN	1.19	3.70	8.20	4.53	2.01
NAN	4.69	3.15	1.14	1.12	7.39	7.89	5.41	1.95
1.88	4.04	4.00	3.62	1.55	4.16	10.10	4.91	2.64
NAN	4.68	3.26	NAN	0.91	3.87	8.11	3.94	1.67
1.32	3.91	3.28	1.91	1.23	6.79	7.75	5.14	2.86
1.07	8.21	3.78	2.80	1.29	7.12	8.50	6.05	2.26

1.41	4.46	3.75	NAN	0.96	3.66	8.05	5.12	1.93
0.89	7.34	3.74	7.96	1.42	7.91	8.76	5.70	1.95
NAN	5.15	3.98	NAN	1.24	4.14	8.68	5.58	2.40
NAN	6.44	3.57	3.60	1.08	4.95	7.90	5.83	2.12
NAN	5.08	3.41	1.35	0.96	6.29	7.66	5.08	1.52
0.88	5.55	3.52	1.30	1.31	4.39	8.47	5.50	1.64
1.11	7.86	3.24	2.55	1.18	7.23	7.76	5.73	3.00
0.99	7.41	2.12	1.02	1.80	7.29	8.78	6.04	1.59
NAN	6.49	3.65	2.05	1.24	7.40	8.24	4.35	1.77
0.94	5.27	3.18	1.49	1.10	5.44	7.79	4.91	2.01
NAN	5.29	2.84	NAN	1.00	5.58	8.27	3.87	1.72
1.30	4.45	3.32	1.04	1.50	7.38	7.87	4.33	2.56
NAN	4.29	3.95	4.96	1.19	7.73	8.94	4.40	2.79
1.05	7.18	3.38	8.34	1.19	7.40	7.87	4.55	1.55
NAN	6.65	3.55	3.46	0.96	7.16	7.66	5.47	3.02
NAN	5.74	3.73	11.90	1.49	7.65	7.56	5.79	2.91
1.05	4.25	2.84	NAN	1.29	3.71	8.45	5.19	1.87
0.92	2.68	4.00	1.28	0.94	3.16	8.06	4.22	1.68
NAN	5.18	3.17	NAN	1.30	3.90	8.66	4.77	1.96
1.10	6.23	3.39	4.18	1.15	5.89	8.15	5.92	2.39
NAN	3.48	3.32	NAN	1.02	3.88	8.83	4.95	2.01
1.03	4.35	3.01	2.86	1.11	3.64	7.34	4.77	1.38
0.93	7.49	4.03	2.82	0.96	7.19	8.53	5.59	2.38
NAN	5.98	1.87	1.36	1.80	7.78	8.02	4.66	1.92
NAN	5.31	3.56	4.62	2.18	8.35	8.40	7.45	2.37
0.83	4.44	4.12	1.57	1.70	7.83	8.26	5.64	2.68
0.96	4.33	3.31	NAN	1.15	3.87	8.70	4.78	1.93
0.93	5.04	3.71	NAN	2.45	7.96	8.09	5.95	2.57
NAN	5.20	2.73	NAN	1.22	2.78	8.10	4.86	1.52
0.85	4.04	3.26	NAN	1.41	6.20	7.45	4.67	2.57
1.13	6.66	3.28	8.65	1.75	7.64	8.57	5.11	3.19
1.24	7.50	3.46	7.07	1.73	7.67	7.66	5.24	2.45
NAN	6.30	3.29	8.29	1.71	7.78	8.00	5.46	2.47
0.82	8.06	3.32	9.89	1.57	8.16	8.81	5.78	2.28
1.21	7.00	3.40	8.40	1.47	7.47	7.89	4.92	2.59
NAN	6.92	3.63	8.32	1.58	7.85	7.84	5.24	1.94
1.10	7.29	3.24	9.04	1.42	7.57	7.93	4.06	2.41
1.34	8.18	3.29	9.13	0.96	7.22	7.15	5.12	1.96
0.83	6.18	3.63	9.97	1.48	7.93	7.80	5.25	2.62
1.06	8.40	3.72	9.00	1.54	7.53	7.85	5.22	2.27

1.44	1.07	0.50	0.27	1.07	1.70	0.47	-0.36	-0.18
154_CXCL5	155_TRANCE	156_HGF	157_IL-12B	161_MMP-10	162_IL-10	164_CCL23	165_CD5	166_MIP-1 alpha
3.44	5.76	8.64	4.24	8.21	1.96	9.24	4.19	0.18
3.77	4.49	9.58	3.99	8.37	NAN	7.95	3.02	0.13
2.64	5.08	8.87	2.39	8.18	NAN	4.99	2.36	-0.06
7.58	5.45	8.91	5.29	7.61	2.13	9.39	4.80	2.60
2.89	4.42	9.58	3.06	7.00	5.66	7.57	3.62	0.08
2.13	4.77	7.94	3.38	7.17	NAN	8.84	2.71	NAN
4.71	4.79	8.85	3.65	7.91	NAN	8.34	3.61	NAN
6.60	5.81	8.58	4.15	6.28	NAN	8.89	3.45	1.71
4.35	4.07	8.98	4.76	8.04	2.91	8.83	5.34	0.16
4.06	5.47	8.83	3.61	7.42	NAN	7.91	3.49	0.18
6.05	5.08	8.54	4.72	7.61	NAN	9.96	4.56	2.72
3.32	5.62	9.42	2.89	7.96	NAN	4.47	2.27	NAN
3.19	4.26	8.56	3.32	7.21	2.44	9.55	3.61	1.58
4.28	5.11	9.44	4.16	8.84	NAN	10.12	3.87	2.06
3.70	4.16	8.82	2.51	7.91	NAN	7.53	2.78	0.08
3.07	4.88	9.25	2.81	9.19	2.05	8.30	2.04	NAN
12.45	5.75	8.03	3.72	8.54	5.58	9.16	3.96	3.58
12.52	6.01	8.32	4.44	9.31	5.08	9.68	3.91	5.71
3.98	4.11	8.30	3.33	7.06	2.37	9.07	3.65	0.30
2.91	5.81	9.16	4.71	7.67	NAN	8.93	3.79	0.25
3.92	5.00	9.87	3.98	8.19	NAN	9.66	3.56	0.70
2.51	5.65	8.90	5.02	8.95	NAN	8.97	4.29	3.32
5.51	5.44	9.19	4.52	8.61	NAN	9.02	3.75	0.16
2.20	5.81	8.59	2.65	7.28	NAN	8.88	2.27	0.99
12.63	5.80	9.09	5.28	7.45	3.88	9.59	4.18	6.86
9.27	4.91	9.31	6.26	9.34	3.49	9.41	4.89	5.94
12.03	6.43	8.61	5.87	7.74	3.88	9.89	4.69	4.58
12.42	5.05	9.18	4.38	9.50	5.03	8.49	4.22	8.72
13.79	6.38	8.35	6.02	9.46	4.36	9.93	4.61	3.47
13.37	5.68	8.60	6.33	10.10	6.99	9.93	5.40	4.38
13.90	6.34	9.07	6.31	9.83	4.88	9.40	5.13	4.78
12.97	4.82	8.96	4.97	10.12	4.36	9.52	4.54	5.88
12.70	6.01	8.26	5.53	8.85	4.28	9.23	4.53	4.79
13.01	3.42	10.14	6.41	10.34	6.21	11.30	4.77	4.49
12.63	5.09	8.71	4.04	7.81	2.75	9.60	3.92	3.15
3.69	6.59	8.93	6.71	7.34	2.00	9.47	4.88	1.51
2.90	6.47	9.20	4.37	8.43	1.98	9.78	5.11	0.80
13.57	4.93	8.86	3.55	7.97	4.33	9.49	3.63	4.10
2.49	5.01	9.18	4.62	9.10	NAN	2.69	4.16	NAN
4.76	4.51	9.20	5.24	7.04	1.96	8.98	4.56	1.37
1.94	4.91	8.97	4.76	8.18	NAN	7.82	3.57	-0.09
3.89	6.60	9.11	4.68	6.67	NAN	8.93	4.23	1.14
4.01	4.45	8.82	2.83	8.25	NAN	8.69	3.47	NAN
10.74	5.45	8.64	4.25	6.91	2.01	8.74	4.21	6.02
4.92	4.85	9.64	4.41	8.41	3.11	7.98	4.56	0.82
3.33	6.00	8.36	3.95	6.67	NAN	8.34	3.84	NAN
5.02	6.21	9.09	5.02	7.95	NAN	9.57	4.49	0.86
12.08	4.97	9.16	4.67	9.02	3.50	9.02	4.16	3.19

3.81	6.04	8.87	3.41	9.06	NAN	8.98	4.04	-0.04
11.30	5.30	9.03	4.28	8.11	2.38	9.84	4.47	7.91
3.56	4.78	9.61	3.51	8.69	NAN	8.74	4.02	-0.04
5.32	5.81	9.07	4.69	8.11	1.94	8.94	4.55	3.44
5.79	4.47	7.91	4.07	7.90	NAN	8.30	4.05	0.72
4.23	6.00	9.21	4.01	9.53	NAN	9.20	4.29	2.01
11.08	5.35	8.62	4.82	7.87	2.56	9.39	4.58	2.47
3.47	5.51	9.53	4.77	9.71	3.47	7.93	4.56	3.87
5.51	5.14	8.85	4.05	6.46	NAN	8.79	4.12	0.57
3.85	5.49	9.05	4.35	9.08	1.97	9.13	4.29	1.22
4.32	6.18	8.42	4.20	7.01	NAN	8.58	4.17	0.20
9.16	4.85	8.99	4.88	7.38	1.77	8.89	4.02	4.44
7.04	5.42	9.63	4.02	7.86	1.71	9.21	4.39	4.27
12.28	6.05	8.86	5.04	9.34	3.45	9.52	4.50	6.94
11.60	5.75	8.91	5.21	7.13	2.75	9.37	4.36	8.07
12.38	5.79	9.41	5.62	6.75	3.70	8.97	4.35	7.11
3.29	5.80	9.41	3.35	8.65	4.20	8.74	3.77	1.19
3.62	4.87	7.88	3.30	7.44	NAN	8.50	3.93	-0.13
3.68	5.03	8.89	3.69	7.92	NAN	8.36	4.01	NAN
6.45	5.31	8.56	3.88	11.38	2.56	9.31	3.99	1.77
2.62	5.31	10.24	2.28	7.46	NAN	6.96	2.99	2.03
3.59	5.16	8.82	3.42	7.95	NAN	7.38	2.99	2.29
11.36	6.06	8.46	4.28	8.29	3.03	9.72	3.86	6.99
2.87	4.94	8.14	4.10	9.14	NAN	9.91	3.69	1.78
11.67	5.07	9.49	6.55	10.23	3.96	10.11	5.62	8.38
6.36	6.05	9.66	4.92	8.80	3.12	10.15	5.40	4.46
2.68	5.34	9.16	3.53	7.57	2.50	8.95	3.65	1.94
7.90	4.20	8.85	4.53	8.50	3.75	9.53	4.96	11.12
2.31	4.80	9.11	3.27	7.59	NAN	9.40	3.03	0.38
4.04	4.51	8.57	4.21	9.23	NAN	9.24	4.02	3.27
12.32	6.01	8.85	5.14	9.49	4.09	9.32	4.00	7.57
12.76	6.20	8.54	5.34	9.11	4.01	9.17	4.42	7.13
12.69	6.31	8.19	4.72	9.53	4.58	9.34	4.69	5.90
12.58	6.80	9.32	5.82	9.36	5.07	9.91	4.88	6.91
12.33	6.01	8.45	4.91	9.38	4.14	8.85	3.93	4.92
11.43	5.35	8.36	4.41	9.73	4.16	9.89	4.50	4.68
13.35	6.12	8.65	5.03	10.84	4.93	9.58	4.74	6.31
11.77	5.96	8.84	4.12	9.56	5.09	8.74	4.08	6.63
12.93	6.76	8.91	5.02	9.98	4.27	9.82	4.56	5.49
12.31	6.02	8.16	5.00	10.86	4.34	9.28	4.23	5.22

1.22	0.69	1.37	1.16	0.11	0.09	0.54	0.88	0.48
167_Flt3L	168_CXCL6	169_CXCL10	172_SIRT2	173_CCL28	174_DNER	175_EN-RAGE	176_CD40	179_FGF-19
9.09	2.33	2.55	1.85	0.27	6.75	8.61	10.56	2.25
8.91	1.95	NAN	1.72	NAN	6.84	8.69	10.22	NAN
8.55	1.19	NAN	1.72	0.12	6.28	8.26	10.65	0.96
9.36	5.27	5.30	NAN	0.22	6.85	7.70	10.49	5.72
8.91	1.57	5.17	NAN	0.21	6.45	9.13	10.54	2.07
9.23	0.77	3.90	NAN	NAN	6.42	6.90	9.53	NAN
9.27	2.33	NAN	NAN	NAN	7.17	8.79	10.31	2.83
9.33	2.84	2.41	1.47	0.19	6.69	8.38	10.50	2.59
8.99	2.26	1.69	NAN	0.11	6.94	8.57	10.63	2.65
9.75	1.98	NAN	1.29	0.30	6.67	8.17	10.90	1.84
9.38	4.18	4.88	1.61	0.22	6.92	8.19	10.82	5.35
7.87	1.27	NAN	NAN	0.19	6.42	8.36	10.24	NAN
9.07	2.53	2.26	1.42	0.17	6.76	7.47	10.40	2.55
8.11	1.78	NAN	1.39	1.13	7.20	8.00	10.83	1.69
7.84	1.23	NAN	1.87	0.20	6.25	8.40	9.98	0.68
8.51	1.02	NAN	NAN	0.13	6.26	8.24	10.73	0.87
10.41	9.06	10.91	5.03	1.87	6.75	7.60	10.68	8.29
9.60	9.44	9.34	4.87	1.45	7.48	7.41	10.12	8.45
7.59	2.18	2.04	NAN	NAN	6.94	8.16	9.93	2.55
9.30	1.48	NAN	NAN	NAN	6.74	8.12	10.81	1.64
9.90	1.66	1.56	NAN	0.23	8.08	8.96	10.91	2.10
9.82	1.47	NAN	1.68	0.19	7.51	7.29	10.40	2.12
9.41	1.85	2.11	1.59	0.76	6.63	8.24	11.07	1.35
8.61	1.28	NAN	NAN	0.18	6.61	7.35	9.96	NAN
9.32	7.75	8.16	5.24	0.55	6.96	8.96	10.48	7.22
10.42	5.88	4.83	3.96	0.72	7.62	9.27	11.13	8.09
9.29	8.25	9.86	4.61	1.07	7.35	7.87	10.14	7.13
9.22	8.54	8.89	3.64	0.94	7.32	6.86	10.14	7.10
9.03	10.06	11.29	4.58	1.37	7.26	6.91	10.10	9.38
9.86	10.25	12.03	3.62	2.71	7.10	7.23	11.01	7.61
9.34	10.23	10.53	4.22	2.40	7.20	5.90	10.60	8.69
8.79	9.28	11.38	5.38	1.44	7.80	7.80	10.16	9.01
9.00	9.42	8.63	6.20	2.10	7.27	7.77	10.17	7.20
8.88	9.73	10.64	5.41	1.61	6.98	6.75	10.94	6.87
8.93	6.31	3.53	3.47	0.58	7.83	7.42	9.92	7.12
9.78	2.60	3.48	2.14	0.54	6.64	8.40	11.32	1.28
9.29	2.26	7.72	NAN	0.18	7.19	8.19	10.59	3.72
8.66	8.45	6.46	4.14	0.63	7.82	7.55	10.87	9.18
9.21	1.15	NAN	NAN	0.34	6.79	7.10	10.67	1.62
8.61	3.10	1.72	1.16	0.48	7.27	8.45	10.54	5.18
8.76	0.71	NAN	NAN	0.20	6.46	7.31	10.24	1.48
8.86	2.12	NAN	NAN	0.32	6.59	8.02	10.66	3.28
8.82	1.09	NAN	NAN	0.14	6.73	7.81	10.15	1.18
8.72	4.69	2.55	NAN	NAN	7.12	7.48	10.31	5.80
9.17	2.56	4.90	1.49	0.35	7.19	8.15	10.40	1.72
9.00	1.71	NAN	NAN	NAN	7.00	7.01	9.56	2.10
8.69	3.68	1.60	1.58	0.20	6.71	7.84	10.60	2.40
8.86	8.10	4.47	1.19	0.51	6.99	7.33	9.91	5.93

8.94	2.05	NAN	1.58	NAN	6.84	8.40	10.60	0.95
8.78	8.41	7.84	1.89	1.01	7.13	6.68	10.33	6.14
8.45	2.35	NAN	1.29	0.25	6.57	8.61	10.74	2.04
9.10	3.67	7.20	1.24	0.71	6.70	8.55	10.21	1.97
9.16	2.58	1.68	NAN	NAN	6.86	6.27	10.13	4.55
9.66	2.04	NAN	NAN	0.39	6.96	8.34	10.99	2.20
8.70	7.32	5.37	1.41	0.24	6.74	7.87	10.00	6.27
9.38	2.32	1.60	1.24	1.00	6.89	8.16	11.11	6.72
8.78	2.62	2.70	2.15	0.16	6.72	7.89	10.72	4.02
8.19	2.11	1.88	1.36	NAN	6.83	8.83	10.57	3.53
9.13	2.65	1.38	NAN	0.30	7.10	7.93	9.70	3.20
8.84	4.05	2.34	2.52	0.22	7.03	7.60	10.14	5.52
9.34	3.68	2.34	3.89	0.51	7.75	8.86	10.97	5.10
8.78	8.93	9.69	4.74	1.80	8.02	7.06	10.16	9.29
8.40	6.39	4.99	3.34	0.17	7.06	7.22	10.01	7.64
8.58	7.08	5.71	4.11	0.70	7.62	8.17	10.25	5.63
8.97	1.60	2.45	1.16	NAN	7.08	8.09	10.86	0.77
9.01	2.01	NAN	1.28	0.40	6.90	7.87	10.32	2.41
8.83	1.11	NAN	1.40	NAN	6.90	8.32	10.46	0.72
9.22	4.86	2.52	NAN	0.30	7.13	6.87	10.22	4.99
7.99	3.05	NAN	1.96	0.19	6.97	8.88	10.81	1.28
8.96	1.71	2.05	1.16	0.30	7.25	8.39	10.48	0.97
8.85	7.51	5.14	1.25	0.41	7.04	7.37	9.97	6.28
8.59	2.21	NAN	1.84	0.93	7.26	7.09	10.01	6.33
10.06	8.08	7.07	2.72	0.75	7.58	8.55	11.36	6.98
8.62	4.78	3.69	4.39	0.78	7.95	8.85	11.13	6.56
8.83	2.44	1.72	1.56	0.27	7.81	8.35	10.64	1.11
9.22	4.56	1.83	1.94	NAN	6.94	8.50	11.62	6.91
9.06	1.45	NAN	1.58	0.25	6.43	7.63	10.58	0.75
9.42	2.41	NAN	1.86	NAN	6.79	7.30	10.60	2.61
8.52	8.84	8.15	5.78	0.64	7.50	6.32	10.45	7.50
8.48	9.11	8.03	4.96	1.08	7.05	6.75	10.14	8.39
9.39	8.93	7.64	7.02	1.26	7.77	5.84	10.18	7.71
9.14	9.18	8.94	5.13	0.99	7.28	5.84	10.26	8.29
8.25	8.73	8.93	4.80	0.85	7.96	7.57	9.83	7.72
9.19	8.98	8.85	4.28	1.60	7.64	6.47	10.01	9.09
9.19	9.44	9.63	5.94	2.09	7.80	7.00	10.25	8.75
8.29	8.14	8.96	5.71	1.30	7.21	6.57	10.27	8.70
9.07	8.80	8.54	5.10	1.28	7.32	7.35	10.20	7.49
9.29	9.59	8.66	5.98	2.61	7.65	6.04	10.71	9.04

1.15	0.43	0.72	1.40	1.46	0.61	0.19	1.04	0.07
183_MCP-2	184_CASP-8	185_CCL25	186_CX3CL1	187_TNFRSF9	188_NT-3	189_TWEAK	190_CCL20	191_ST1A1
8.24	2.09	NAN	4.40	6.65	NAN	5.69	6.87	NAN
4.33	1.77	NAN	1.87	5.98	0.84	4.14	1.81	NAN
4.72	1.82	1.10	3.06	5.63	0.64	4.29	2.86	NAN
9.24	3.02	1.38	6.28	6.62	NAN	6.04	10.24	0.20
3.35	0.68	4.37	1.78	5.97	0.62	4.16	12.62	0.81
6.03	0.57	NAN	3.16	5.29	NAN	4.74	3.58	NAN
6.79	2.43	1.08	4.21	5.64	NAN	5.02	6.79	NAN
7.88	2.64	1.08	3.45	6.13	0.71	5.40	6.38	NAN
7.89	1.63	NAN	4.84	7.10	NAN	5.06	10.27	NAN
3.39	2.03	NAN	3.77	6.33	NAN	6.32	5.74	0.20
8.24	3.51	1.48	4.99	6.44	0.69	5.56	6.15	0.73
4.07	NAN	NAN	1.45	5.67	0.64	4.40	2.48	NAN
7.65	1.98	1.32	4.22	5.61	NAN	4.32	9.90	0.36
7.72	2.28	1.27	4.58	6.32	1.07	5.01	7.01	NAN
4.64	1.21	NAN	2.37	5.18	NAN	4.36	2.02	NAN
4.96	0.89	0.91	2.50	6.65	NAN	4.29	10.47	NAN
11.39	6.46	6.32	7.98	6.47	1.76	8.16	8.00	3.42
10.11	5.27	6.59	7.18	6.25	1.83	8.81	5.82	3.13
6.98	1.56	NAN	4.74	5.69	NAN	5.57	8.63	NAN
5.72	1.65	NAN	3.04	6.76	0.74	5.12	5.85	NAN
5.14	1.66	1.86	3.78	6.65	1.15	5.58	4.61	NAN
7.14	2.71	NAN	5.18	6.87	0.81	5.18	11.83	NAN
8.14	2.48	1.16	4.18	7.13	0.78	5.17	7.83	0.57
4.16	1.25	NAN	2.25	5.44	1.08	3.83	2.42	NAN
10.68	8.22	5.15	6.98	7.10	1.66	7.44	7.66	3.63
8.88	9.02	1.58	6.61	7.99	NAN	6.41	8.81	2.82
8.81	7.12	4.56	7.09	7.27	1.58	8.10	8.24	3.94
10.38	4.63	6.60	6.82	6.55	1.67	8.52	6.71	4.37
11.06	4.73	5.44	6.59	7.31	1.44	8.78	6.95	2.07
11.13	4.03	6.47	7.65	8.91	2.70	8.74	12.23	1.68
10.51	5.63	4.84	6.98	8.13	3.47	9.05	6.38	3.33
8.59	5.10	6.64	7.91	7.21	1.84	9.17	7.05	4.81
10.49	5.22	6.13	7.06	7.04	2.03	9.04	6.53	4.77
9.89	4.95	6.30	8.38	7.10	2.66	8.47	10.94	5.09
10.04	5.86	2.07	7.47	6.24	2.00	7.39	7.09	1.16
8.74	2.61	1.33	4.73	9.19	0.69	4.69	7.77	0.83
6.84	3.40	NAN	4.54	6.32	NAN	5.46	6.92	NAN
9.95	5.83	7.06	7.17	6.24	1.80	8.10	6.54	1.99
6.37	0.73	0.94	NAN	6.07	2.75	5.01	6.19	NAN
7.80	4.04	1.48	4.80	6.51	0.95	6.39	6.82	0.71
5.19	0.47	0.81	NAN	6.79	0.61	4.02	4.46	NAN
8.38	2.44	0.87	3.93	6.60	NAN	5.20	6.76	NAN
5.34	1.32	0.85	3.42	6.27	0.84	4.56	5.92	NAN
9.62	3.59	1.58	5.95	6.41	NAN	5.77	7.12	0.53
7.91	2.41	1.04	4.63	6.96	0.94	4.96	7.55	NAN
6.17	1.19	0.85	4.65	6.04	NAN	4.41	8.82	0.20
8.32	3.19	NAN	4.00	6.80	0.71	6.48	6.99	0.41
10.45	4.59	1.86	5.58	5.86	NAN	6.46	7.26	0.52

7.10	2.58	1.01	4.14	6.12	NAN	5.35	6.40	1.76
10.16	6.89	3.10	6.11	6.71	NAN	8.06	7.74	1.72
3.24	1.70	NAN	3.45	6.10	1.58	5.00	6.13	NAN
7.69	2.81	NAN	4.42	6.32	NAN	5.36	8.71	0.40
8.86	2.12	1.34	4.91	6.45	NAN	4.88	5.81	1.73
8.52	2.44	1.61	4.02	6.66	0.75	5.51	8.29	3.12
9.38	3.97	4.84	5.84	6.36	NAN	6.09	8.63	1.18
8.54	3.18	0.82	3.52	6.82	NAN	5.74	10.19	0.96
8.77	5.03	2.53	4.42	6.45	0.69	5.91	5.83	0.92
9.19	2.61	NAN	3.77	6.26	0.65	5.62	7.83	0.08
7.22	1.63	NAN	5.16	6.14	NAN	5.30	9.30	0.52
3.98	5.47	2.76	5.73	6.49	0.72	6.04	6.17	0.36
8.79	6.09	2.60	5.78	6.40	1.35	6.91	8.68	1.16
10.25	6.73	3.56	6.89	6.36	2.61	8.92	6.69	5.25
8.28	5.87	2.79	6.55	6.46	1.51	7.53	8.61	1.96
9.12	6.55	3.99	6.20	6.87	2.24	7.81	9.07	2.34
6.05	1.62	0.96	3.09	6.94	1.08	4.43	6.20	NAN
6.37	1.42	1.14	4.68	6.05	0.67	4.53	4.34	0.08
5.98	1.31	NAN	3.53	6.22	NAN	4.46	5.22	NAN
9.64	2.19	1.25	4.67	6.38	0.76	6.14	7.68	1.02
5.06	1.97	1.21	3.27	6.12	NAN	4.78	5.16	NAN
3.93	1.41	1.06	1.93	6.43	1.48	4.46	4.21	NAN
10.22	4.61	1.97	6.09	5.81	NAN	6.13	8.18	1.23
8.71	1.90	1.04	4.61	6.23	NAN	6.50	6.65	0.76
12.73	7.09	2.14	7.38	8.68	0.96	7.11	8.88	1.71
8.89	7.98	1.79	5.42	7.35	1.80	7.47	7.17	2.53
6.26	2.28	1.29	4.07	6.24	1.09	5.29	6.60	NAN
8.73	4.95	NAN	6.32	8.02	NAN	5.90	12.39	0.84
4.41	1.68	0.86	2.72	6.28	0.67	4.38	3.39	NAN
6.96	2.98	NAN	5.94	6.62	0.80	5.52	7.69	NAN
10.76	5.05	5.88	6.29	6.69	1.22	8.82	7.14	2.18
10.09	5.53	5.79	7.50	6.23	1.72	8.66	6.11	4.02
9.68	5.06	7.07	6.95	6.96	1.52	8.97	5.72	3.88
10.60	5.49	6.05	6.75	7.13	1.40	9.05	6.41	4.84
9.88	4.96	5.92	6.89	6.56	1.41	9.29	4.97	5.14
10.51	5.06	6.57	6.54	6.74	2.28	9.11	7.81	5.02
11.03	4.75	7.46	6.93	6.95	2.40	9.46	6.88	5.90
11.11	4.96	6.33	6.85	6.48	1.41	8.70	9.39	6.12
10.18	5.15	5.83	6.70	6.78	1.78	9.32	6.24	6.43
9.55	4.66	7.66	6.66	6.80	2.26	9.51	7.38	5.46

0.96	0.90	0.33	0.15	
192_STAMPB	194_ADA	195_TNFB	196_CSF-1	#Chip ID.
1.74	3.33	0.57	8.61	1
2.15	3.24	NAN	8.23	1
2.40	2.23	NAN	7.91	1
1.69	3.58	1.37	8.43	1
NAN	3.92	NAN	8.75	1
NAN	3.11	NAN	8.34	1
1.49	3.54	0.35	8.27	1
1.10	3.71	NAN	7.93	1
1.56	4.09	0.34	8.77	1
1.72	3.69	NAN	8.35	1
2.63	3.10	1.02	8.64	1
1.22	2.89	NAN	7.51	1
1.57	3.26	NAN	8.67	1
2.20	3.73	NAN	8.13	1
1.95	3.43	NAN	7.63	1
1.38	3.75	NAN	8.42	1
4.23	5.66	4.11	8.63	2
3.71	4.58	3.53	8.42	1
1.41	3.79	NAN	8.00	1
1.74	3.45	NAN	8.33	1
1.98	4.53	NAN	8.77	1
1.95	3.90	0.66	8.77	1
2.31	4.27	0.50	8.29	1
2.02	3.72	2.74	7.64	1
4.90	5.42	4.14	8.40	1
4.94	5.76	1.45	8.80	1
3.93	4.93	4.83	8.43	1
3.15	4.49	3.57	8.43	1
3.48	4.63	4.12	8.67	1
3.55	5.58	4.18	8.71	1
3.53	5.25	3.85	8.66	1
4.36	5.29	3.69	8.68	1
4.86	5.07	4.50	8.48	1
3.82	4.92	3.46	9.03	1
3.70	4.21	2.96	8.14	1
2.86	3.45	1.64	9.03	1
1.37	4.26	0.38	9.00	1
3.89	4.60	3.77	8.31	1
NAN	4.24	NAN	8.38	1
0.97	4.23	0.37	8.64	1
NAN	2.46	NAN	8.67	1
1.50	3.34	NAN	8.43	1
1.63	3.39	NAN	8.28	1
2.13	4.01	0.56	8.19	1
2.33	2.48	0.90	8.81	1
1.60	2.69	7.96	8.30	1
2.35	4.17	0.45	8.41	1
3.43	3.48	3.01	8.08	1

2.69	3.73	0.50	8.14	1
3.27	4.45	2.57	8.35	1
2.09	3.55	NAN	8.11	1
2.46	3.30	1.76	8.18	1
1.93	3.24	1.42	8.22	1
2.04	3.01	0.62	8.59	1
3.12	4.08	1.94	8.28	1
1.61	4.20	NAN	9.08	1
3.16	4.81	1.21	8.31	1
2.03	4.40	3.45	8.18	1
1.49	2.87	8.11	8.17	1
2.76	4.07	1.99	8.31	1
3.79	4.95	2.46	8.42	1
3.95	5.27	3.64	8.34	1
3.44	4.41	3.47	7.99	1
4.21	4.80	3.71	8.30	1
1.86	3.36	NAN	8.43	1
1.79	3.06	0.42	8.06	1
1.72	4.10	NAN	8.65	1
2.08	3.71	0.96	8.31	1
1.87	4.12	NAN	8.49	1
2.62	3.94	0.45	8.02	1
2.95	4.02	3.16	7.93	1
4.09	4.41	NAN	8.67	1
4.10	5.42	1.89	8.91	1
5.83	6.45	2.37	8.66	1
2.51	4.54	NAN	8.34	1
2.19	4.34	0.86	8.96	1
2.50	3.76	NAN	8.54	1
2.29	3.87	NAN	8.25	1
4.25	5.26	4.05	8.52	1
4.13	5.06	3.68	8.01	2
5.54	5.29	3.77	8.30	1
3.81	5.00	3.83	8.52	1
3.65	5.59	3.53	8.20	1
3.33	5.04	3.69	8.18	1
3.86	5.22	4.62	8.39	1
3.77	4.70	3.89	7.90	2
3.57	4.52	3.84	8.32	1
4.16	5.19	3.91	7.99	2
