# Supplementary material for: Correlation of serum cytokines, chemokines, growth factors and enzymes with periodontal disease parameters
Source: PLoS One. 2017 Nov 30;12(11):e0188945. doi: 10.1371/journal.pone.0188945 (PMC5708747; doi:10.1371/journal.pone.0188945)
Supplement: S2 Table — NA = Not available. NA data cells were treated as zero in all calculations. *R = Rheumatoid arthritis (green), P = Periodontal disease (orange), H = Healthy (grey). (PDF) [file pone.0188945.s003.pdf]

Subject ID	BOP	PPD 3-<5mm	PPD ≥5mm	MBL	Number of Teeth
R1	46.4	44	0	2.05	28
R2	70.5	32	0	2.29	28
R3	40	84	0	2.73	28
R4	57	55	0	4.4	19
R5	21	27	0	3.3	27
R6	85	57	0	2.3	28
R7	25	55	0	3.36	28
R8	94	64	0	3.1	28
R9	5	68	2	4.42	28
R10	77	69	0	3.09	27
R11	60	68	0	1.47	28
R12	44	55	0	2.25	28
R13	90	70	0	2.58	28
R14	54	47	0	2.54	28
R15	16	55	0	4.28	26
R16	7	41	0	3.67	18
R17	5	45	2	2.93	28
R18	10	35	2	NA	28
R19	5	84	0	3.55	28
R20	77	76	5	7.17	26
R21	59	73	3	4.57	28
R22	68	50	7	4.52	22
R23	88	58	23	3.94	28
R24	33	72	7	3.98	28
R25	94	24	36	4.57	27
R26	31	31	27	5.12	22
R27	45	28	17	3.4	25
R28	19	43	16	5.83	28
R29	3	33	3	NA	22
R30	21	21	5	5.83	11
R31	26	44	13	4.2	25
R32	3	47	3	NA	28
R33	34	36	21	NA	27
R34	2	31	21	NA	28
R35	5	25	43	4.64	28
R36	82	31	29	3.26	27
R37	5	68	9	5.12	27
R38	4	35	58	5.4	27
P1	100	50	17	4.9	19
P2	24	51	29	5.5	23
P3	95	78	15	10.89	28
P4	38	15	32	7.63	12
P5	91	31	32	5.93	16
P6	79	62	25	10.1	28
P7	92	40	29	8.96	19
P8	97	60	28	3.65	23
P9	100	64	40	4.54	27
P10	77	64	31	3.8	28
P11	83	72	32	2.75	28
P12	100	57	22	5.52	26
P13	98	48	52	4.52	26
P14	38	16	36	4.6	27
P15	100	50	26	4.3	26
P16	78	72	41	3.88	27
P17	93	54	41	3.5	26
P18	100	55	37	4.89	23
P19	92	43	50	5.53	28
P20	100	30	40	4.42	26
P21	90	27	55	4.44	27
P22	35	26	51	4.5	28
P23	100	38	48	3.75	26
P24	76	50	52	3.065	28
P25	100	41	57	4.68	28
P26	73	43	26	6.6	27
P27	98	62	49	12.7	28
P28	75	40	11	7.8	15
P29	96	9	47	7.52	14
P30	100	53	33	NA	24
P31	12	73	9	5.24	25
P32	36	53	5	NA	26
P33	41	58	22	5.61	27
P34	43	65	43	7.695	28
P35	34	52	26	5.57	26
P36	100	30	50	5.94	27
P37	54	62	10	4.02	26

P38	56	77	20	NA	28
H1	44	36	1	4.41	27
H2	100	18	1	4.97	14
H3	100	19	0	3.47	16
H4	53	15	1	3.05	28
H5	74	22	0	2.33	28
H6	48	18	0	3.26	28
H7	100	22	0	2.55	28
H8	5	27	1	3	28
H9	65	16	0	3.11	28
H10	94	38	0	2.95	28
H11	88	20	1	2.88	28
H12	100	16	0	3.2	28
