# Supplementary material for: Correlation of serum cytokines, chemokines, growth factors and enzymes with periodontal disease parameters
Source: PLoS One. 2017 Nov 30;12(11):e0188945. doi: 10.1371/journal.pone.0188945 (PMC5708747; doi:10.1371/journal.pone.0188945)
Supplement: S3 Table — Significant results (p-value < 0.05) are in blue while correlations ≥ 0.05 are shown in black. (PDF) [file pone.0188945.s004.pdf]

	BOP	PPD 3-<5mm	PPD >5mm	MBL	Number of Teeth
101_IL-8	0.164	-0.108	0.201	0.128	0.027
102_VEGF-A	-0.077	-0.099	-0.025	-0.154	0.296
105_MCP-3	0.130	-0.266	0.071	-0.010	0.163
107_CDCP1	0.157	0.002	0.225	0.366	-0.533
108_CD244	0.283	-0.294	0.226	0.148	0.075
109_IL-7	0.142	-0.281	0.052	-0.151	0.267
110_OPG	0.030	0.166	0.032	0.113	-0.213
111_LAP TGF-beta-1	0.121	-0.386	0.003	-0.093	0.239
112_uPA	0.123	-0.128	0.122	-0.058	0.184
113_IL-6	0.202	0.036	0.215	0.114	-0.038
115_MCP-1	0.169	-0.185	0.019	-0.140	0.160
117_CXCL11	0.117	-0.355	-0.078	-0.180	0.314
120_TRAIL	0.034	-0.292	0.082	-0.129	0.218
122_CXCL9	0.062	-0.130	0.119	0.015	0.175
123_CST5	0.152	-0.208	0.109	0.013	-0.016
126_OSM	0.098	-0.226	0.126	0.049	0.068
128_CXCL1	0.177	-0.311	0.113	-0.077	0.170
130_CCL4	0.008	-0.303	-0.015	-0.064	0.295
131_CD6	0.231	-0.281	0.292	0.246	-0.088
132_SCF	0.037	-0.361	0.004	-0.109	0.226
133_IL-18	0.031	-0.181	-0.079	-0.066	0.018
134_SLAMF1	0.048	-0.314	0.082	-0.023	0.025
135_TGFA	0.024	-0.303	0.076	-0.080	0.264
136_MCP-4	0.092	-0.221	0.203	-0.043	0.229
117_CXCL11	0.117	-0.349	-0.078	-0.180	0.311
138_TNFSF14	0.110	-0.219	0.163	-0.016	0.207
140_IL-10RA	0.152	-0.127	-0.063	-0.266	0.069
142_MMP-1	0.066	-0.272	0.030	-0.232	0.269
143_LIF-R	0.071	-0.135	0.329	0.119	-0.018
145_CCL19	0.116	-0.377	-0.025	-0.162	0.183
148_IL-15RA	-0.008	-0.146	-0.093	0.011	0.107
149_IL-10RB	0.060	-0.284	0.155	0.013	0.137
151_IL-18R1	-0.021	0.120	0.117	0.233	-0.255
152_PD-L1	-0.019	0.042	0.117	-0.063	0.068
153_Beta-NGF	0.114	-0.248	-0.007	-0.144	0.147
154_CXCL5	0.064	-0.393	-0.008	-0.185	0.275
155_TRANCE	0.208	-0.314	0.101	-0.111	0.157
156_HGF	0.015	0.001	0.232	0.305	-0.244
157_IL-12B	0.183	-0.328	0.153	0.103	0.110
161_MMP-10	0.141	-0.273	-0.089	-0.251	0.113
162_IL-10	-0.065	-0.320	-0.133	-0.178	0.267
164_CCL23	0.017	-0.135	0.046	-0.148	0.250
165_CD5	0.174	-0.255	0.311	0.142	-0.006
166_MIP-1 alpha	0.057	-0.265	0.046	-0.088	0.201
167_Flt3L	0.020	-0.036	-0.097	-0.198	-0.010
168_CXCL6	0.050	-0.311	0.008	-0.180	0.274
169_CXCL10	0.010	-0.357	-0.115	-0.248	0.343
172_SIRT2	0.017	-0.352	-0.284	-0.340	0.378
173_CCL28	0.127	-0.406	0.053	-0.005	0.157
174_DNER	-0.450	0.035	-0.679	-0.512	0.401
175_EN-RAGE	-0.381	0.062	-0.594	-0.463	0.349
176_CD40	-0.423	0.353	-0.253	-0.214	0.048
179_FGF-19	0.387	-0.127	0.550	0.459	-0.354
183_MCP-2	-0.174	-0.132	-0.396	-0.449	0.513
184_CASP-8	0.342	-0.341	0.310	0.148	-0.002
185_CCL25	0.134	-0.415	0.327	0.075	0.212
186_CX3CL1	-0.252	-0.142	-0.569	-0.500	0.497
187_TNFRSF9	-0.211	-0.244	-0.479	-0.446	0.436
188_NT-3	0.379	-0.331	0.379	0.303	-0.078
189_TWEAK	-0.323	0.016	-0.632	-0.522	0.358
190_CCL20	-0.093	-0.227	-0.257	-0.216	0.278
191_ST1A1	0.410	-0.190	0.591	0.334	-0.255
192_STAMPB	0.490	-0.157	-0.367	-0.499	0.502
194_ADA	0.042	-0.376	0.052	-0.038	0.135
195_TNFB	0.146	-0.411	0.110	-0.177	0.196
196_CSF-1	-0.055	0.079	0.121	0.197	-0.174
